# Overlapping expression characteristics of ubiquitination-related genes in periodontitis and renal cell carcinoma: transcriptomic analysis and experimental validation

**DOI:** 10.1186/s12903-026-08574-2

**Published:** 2026-05-19

**Authors:** Xiaofeng Guo, Peiyao Liu, Lijuan Zhang, Yuxin Zhang, Wenbo Zhai, Weilun Zhang, Chengzhi Zhang, Dong Liu, Jing Shi, Lei Pang

**Affiliations:** 1 Department of Stomatology, Shanxi Provincial People’s Hospital, Taiyuan, Shanxi 030012 China; 2 Shanxi Oral Health Prevention and Control Technology Innovation Center, Taiyuan, Shanxi 030012 China; 3 Department of Urology, Shanxi Provincial People’s Hospital, Taiyuan, Shanxi 030012 China

**Keywords:** Renal cell carcinoma, Periodontitis, Ubiquitination, Transcriptomic analysis, Biomarkers, Therapeutic targets, Expression validation

## Abstract

**Objective:**

Through transcriptomic analysis and expression level validation, ubiquitin-related genes with overlapping expression profiles between renal cell carcinoma and periodontitis were identified.

**Materials and methods:**

RNA-seq datasets of renal cell carcinoma from The Cancer Genome Atlas (TCGA) and periodontitis from the Gene Expression Omnibus (GEO) were analyzed to identify differentially expressed genes (DEGs) in each disease. A total of 322 ubiquitination-related genes were integrated with the identified DEGs to screen ubiquitination-related genes with overlapping expression patterns between renal cell carcinoma and periodontitis. Survival analysis, functional enrichment analysis, protein–protein interaction (PPI) network construction, and subsequent expression validation via quantitative real-time PCR (qPCR) and immunohistochemistry (IHC) were performed on these overlapping genes in clinical samples.

**Results:**

In this study, differentially expressed genes (DEGs) in renal cell carcinoma and periodontitis were screened by analyzing the TCGA-KIRC dataset and periodontal disease datasets from the GEO database. Meanwhile, 332 ubiquitination genes collected from published literature were intersected separately with DEGs of renal cell carcinoma and periodontitis. Subsequent Venn diagram analysis identified 10 co-regulated ubiquitination-related genes (ALDOB, FABP5, IFI16, IKZF1, LGALS1, LSP1, MNDA, RPL13, VIM, WAS) as crosstalk genes between renal cell carcinoma and periodontitis. To systematically screen the genes with the strongest comprehensive regulatory capacity, the above 10 crosstalk genes were comprehensively evaluated in parallel from two independent and critical dimensions. In terms of the shared molecular mechanisms underlying periodontitis and renal cell carcinoma, six genes (IKZF1, LGALS1, LSP1, MNDA, VIM and WAS) were consistently upregulated in both disease tissues, indicating their potential involvement in the common pathogenesis of the two diseases. Tumor mutation analysis further indicated that the above 10 URGs were significantly correlated with the tumor immune microenvironment. Functional enrichment analysis revealed that these genes may affect disease progression via ubiquitin-mediated proteolysis and the p53 signaling pathway.With regard to the correlation between gene expression levels and survival outcomes of patients with renal cell carcinoma, subsequent Kaplan–Meier survival analysis verified that among the 10 co-regulated ubiquitination-related genes, six genes (ALDOB, FABP5, IFI16, LGALS1, RPL13, WAS) exhibited significant correlations with the prognosis of RCC.LASSO regression was subsequently performed to screen the four genes (ALDOB, IFI16, WAS, IKZF1) that showed significance in multivariate regression analysis. Finally, WAS and IKZF1 were identified as the key ubiquitination regulatory genes underlying renal cell carcinoma and periodontitis.Consistent high expression levels of WAS and IKZF1 in both periodontitis and renal cell carcinoma tissues were verified by qPCR and immunohistochemistry (IHC), compared with normal control tissues. The protein–protein interaction (PPI) network and miRNA-prognostic gene regulatory network were constructed using Cytoscape software.

**Conclusion:**

This study firstly reveals that WAS and IKZF1 are ubiquitin-related genes with overlapping expression patterns in renal cell carcinoma and periodontitis, suggesting that they may participate in the occurrence and progression of the two diseases by regulating ubiquitination-related pathways. This finding not only enriches our understanding of the molecular expression characteristics of renal cell carcinoma and periodontitis but also verifies the potential of WAS and IKZF1 as cross-disease targets. It provides novel insights into the screening of potential biomarkers for these two diseases, and offers preliminary references for further exploring the clinical translational value of ubiquitination-related genes in renal cell carcinoma and periodontitis.

**Clinical trial number:**

Not applicable.

**Graphical Abstract:**

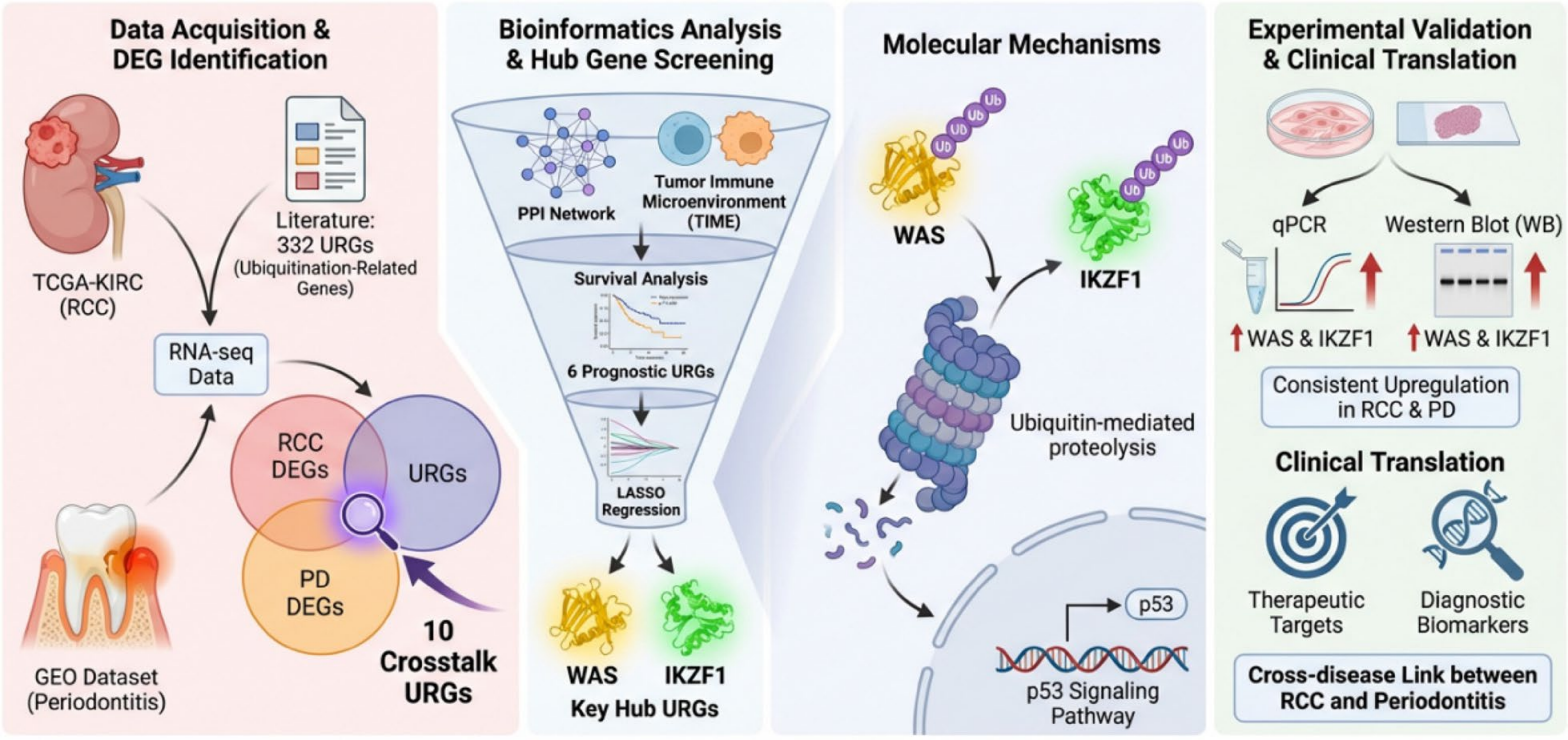

**Supplementary Information:**

The online version contains supplementary material available at 10.1186/s12903-026-08574-2.

## Introduction

Periodontitis (PD), as a common chronic inflammatory oral disease, is mainly characterised by progressive destruction of the periodontal supporting tissues and pathological absorption of the alveolar bone, ultimately leading to tooth loosening and loss, severely affecting the patient’s oral function and quality of life [1–4]. Periodontitis is not an isolated local oral lesion [5]; The inflammatory factors produced by periodontitis can enter the whole body through the bloodstream, participating in and regulating the immune microenvironment and pathophysiological changes of corresponding distant organs, and it has a certain correlation with various systemic diseases such as diabetes and cancer [6, 7].Its pathological processes, including chronic inflammation-mediated immune disorders, oxidative stress and abnormal microbial metabolism may share potential common regulatory mechanisms with the occurrence and progression of malignant tumors [8] .Meanwhile, renal cell carcinoma (RCC) is one of the common malignant tumours of the urinary system worldwide, with persistently high incidence and mortality, especially with a poor prognosis in advanced patients [9, 10]. A breakthrough has been made in the diagnosis and treatment of RCC, but there is still much room for in-depth exploration in the pathogenesis field. Epidemiological studies have suggested a potential association between periodontitis and renal cell carcinoma [11–13]. Nevertheless, the available evidence remains insufficient; definitive clinical correlation evidence has not yet been established, and the underlying molecular mechanisms linking the two diseases remain unclear.

Ubiquitination, a core mechanism of post-translational protein modification, plays a critical role in regulating key biological processes such as cell cycle progression, inflammatory responses, and immune modulation [14].Recently, in the cancer and inflammatory diseases field, ubiquitination-related genes have served as a research focus [15, 16].But their specific mechanisms in the crosstalk regulation between RCC and periodontitis are still in terra incognita.Accordingly, systematic mining and validation of abnormally expressed ubiquitination-related genes in RCC and periodontitis provide preliminary clues for exploring the potential common pathological mechanisms of the two diseases and identifying potential diagnostic and therapeutic targets, which possess considerable scientific significance.

Ubiquitination-specific assays and functional experiments were not performed in the present study. This study aimed to explore the expression profiles and potential regulatory relationships of ubiquitination-related genes in RCC and periodontitis by integrating multi-omics data.We made use of transcriptome data from the databases of TCGA and GEO and combined such data with the analysis of differential expression, the analysis of survival, and the analysis of functional enrichment to find out genes that are key and related to ubiquitination, and we carried out experiments to confirm their expression patterns present in samples of clinical nature.This study not only provides novel insights into understanding the molecular association between RCC and periodontitis, but also offers preliminary theoretical references for the development of biomarkers and targeted therapeutic strategies for related diseases. It is expected to provide valuable explorations for interdisciplinary research on inflammation and cancer.


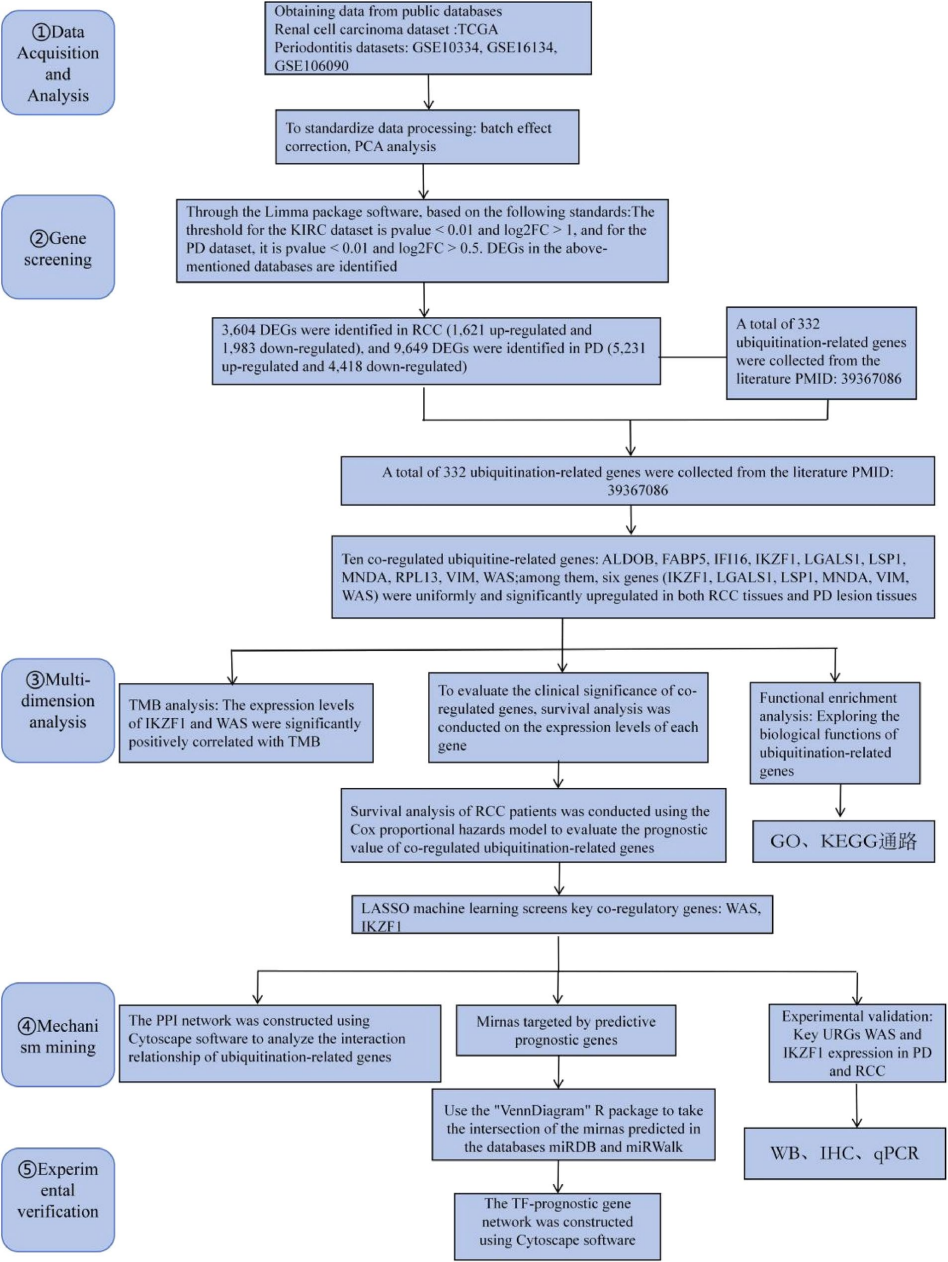


## Results

### Data processing

**Fig. 1:**
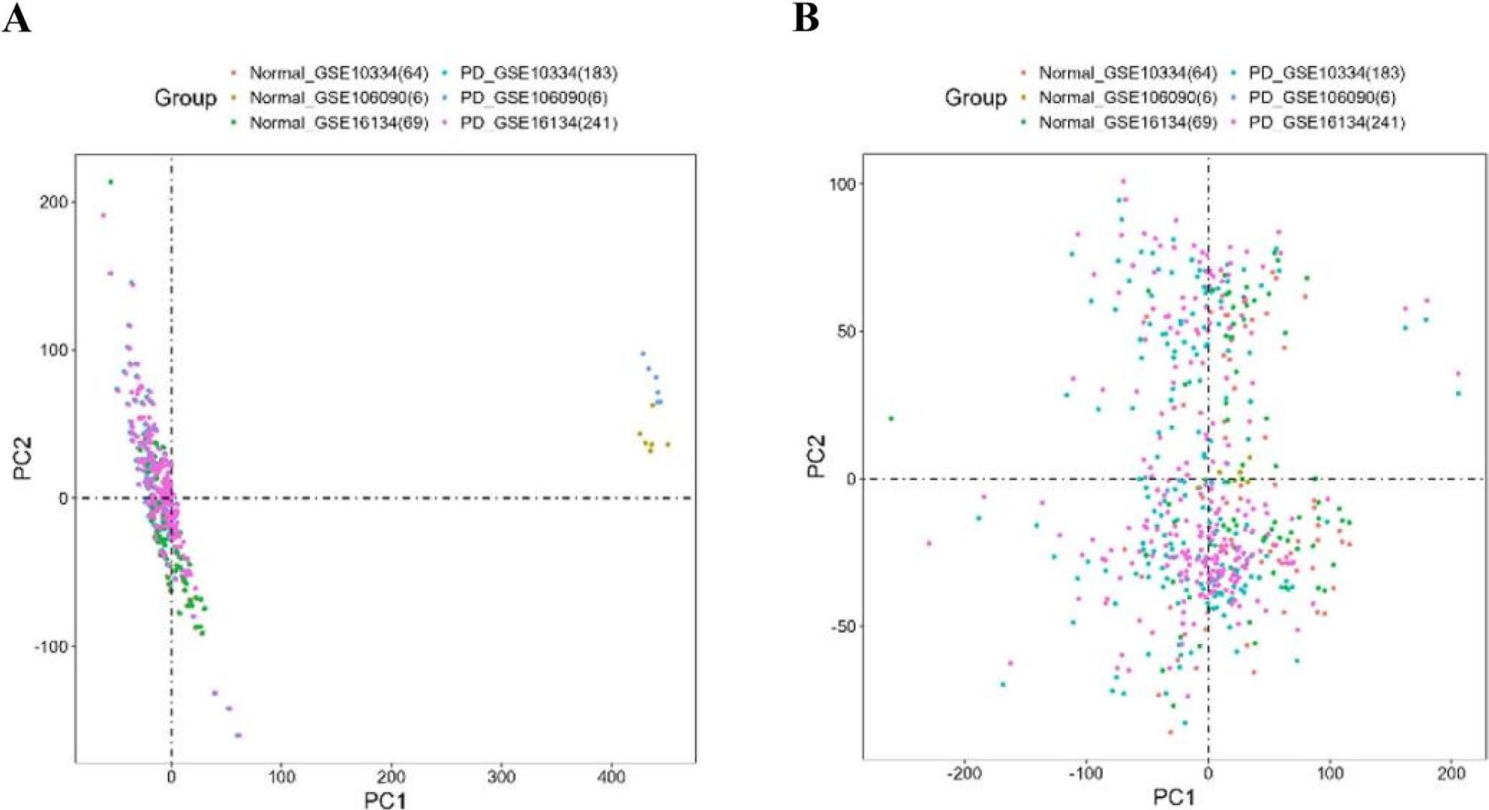
Data distribution of periodontitis (PD) datasets before and after batch effect correction. **A **PD datasets before batch effect removal. **B** PD datasets after batch effect removal. Figure legend:Batch effect removal reduced technical variation among samples, resulting in more concentrated and consistent data distribution, which provided a reliable basis for subsequent differential expression analysis

To ensure the reliability of subsequent analyses, strict quality control and normalization were first performed on raw expression data.As for the periodontitis dataset, we carried out a merging action on three datasets that are independent: GSE10334, GSE16134, and GSE106090(https://www.ncbi.nlm.nih.gov/geo/). Since different research platforms and experimental batches can bring in systematic variation, we carried out an inter - sample normalisation by making use of the `normalizeBetweenArrays` function, which comes from the `limma` package, and put the `ComBat` algorithm into use to correct the batch effects.As shown in Fig. 1, samples were clearly clustered according to dataset origin before batch effect removal (Fig. 1A). After batch correction, sample distributions across different datasets became homogeneous, and technical variation was significantly reduced (Fig. 1B), which provides a reliable basis for subsequent differential expression analysis.

**Fig. 2:**
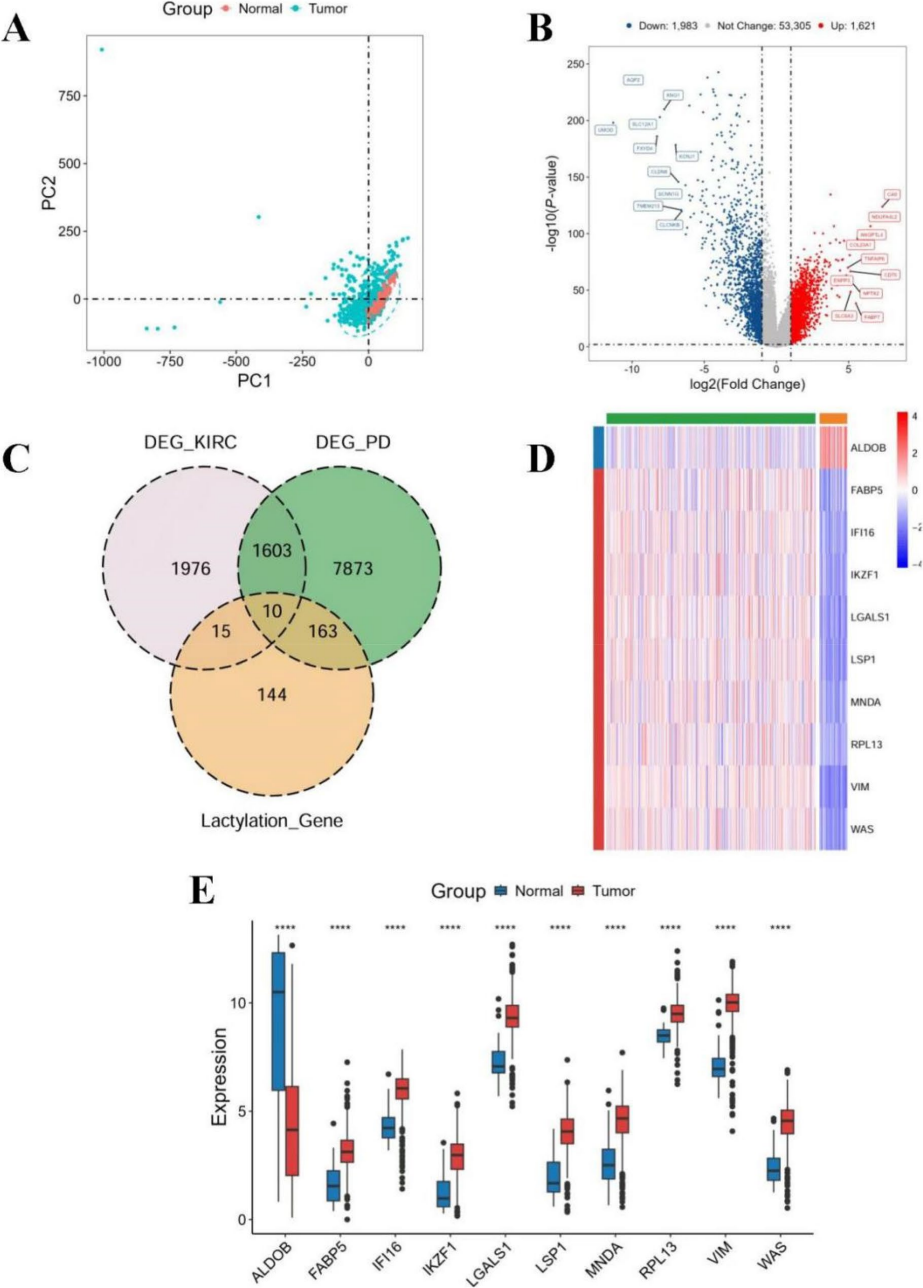
Screening analysis of ubiquitination-related genes. **A** Principal component analysis (PCA) of the TCGA-KIRC dataset. **B** Volcano plot of differentially expressed genes (DEGs) in RCC. **C** Venn diagram of ubiquitination-related genes, RCC DEGs and PD DEGs. **D** Heatmap showing expression levels of the 10 overlapping URGs in RCC samples. **E** Boxplot showing expression levels of the 10 overlapping URGs in RCC samples

To evaluate transcriptomic heterogeneity in renal cell carcinoma samples, principal component analysis (PCA) was performed on the TCGA-KIRC dataset (https://genome-cancer.ucsc.edu/).PC A analysis confirmed the presence of heterogeneity between RCC tumor and normal samples, and provided a basis for subsequent screening of differentially expressed genes.As shown in Fig. 2A, tumor and normal control samples were clearly separated along the PC1 axis, indicating global differences in gene expression between the two groups. This result further supports the identification of RCC-related differentially expressed genes. Batch effect removal is a critical step in data analysis, which significantly improves data comparability and the accuracy of analytical results.

### Differential gene expression analysis

Based on normalized gene expression data, differential expression analysis was performed on RCC and periodontitis samples, respectively. Using the limma package, we identified 3,604 differentially expressed genes (DEGs) in RCC, including 1,621 upregulated and 1,983 downregulated genes. For periodontitis, a total of 9,649 DEGs were identified, consisting of 5,231 upregulated and 4,418 downregulated genes.To better visualize the distribution of differentially expressed genes in RCC and periodontitis, volcano plots were generated using the ggplot2 package, as shown in Fig. 2B.In the DEG volcano plots for RCC and periodontitis, significant points on both sides represent upregulated and downregulated DEGs in the two diseases. These findings indicate extensive transcriptomic dysregulation in both RCC and periodontitis, and provide a basis for subsequent screening of shared key regulatory genes between the two diseases.

### Screening of ubiquitination-related genes and expression patterns

To explore the potential role of ubiquitination-related genes in the comorbidity mechanism of RCC and periodontitis, 332 ubiquitination-related genes (URGs) systematically collected from the literature were overlapped with the differentially expressed genes of RCC and periodontitis, respectively. As shown in Fig. 2C, Venn diagram analysis identified 10 ubiquitination-related genes that may participate in the regulation of comorbidity: ALDOB, FABP5, IFI16, IKZF1, LGALS1, LSP1, MNDA, RPL13, VIM, WAS.

Further analysis of the expression profiles of these 10 genes in RCC revealed by heatmaps (Fig. 2D) and boxplots (Fig. 2E) that six genes (IKZF1, LGALS1, LSP1, MNDA, VIM, WAS) were consistently and significantly upregulated in both RCC tumor tissues and periodontitis lesions. This observation demonstrates shared ubiquitination regulatory characteristics between PD and RCC at the molecular level.These findings indicate that a specific panel of ubiquitination-related genes exhibits consistent dysregulation in RCC and PD, suggesting potential shared molecular mechanisms between the two diseases rather than completely independent pathological processes.The present study provides preliminary evidence that URGs may be involved in the cross-pathological mechanisms underlying inflammation and cancer, as well as candidate targets and a research basis for further investigation into ubiquitination-mediated comorbidity mechanisms.

### Tumour mutation burden and gene mutation landscape analysis

**Fig. 3:**
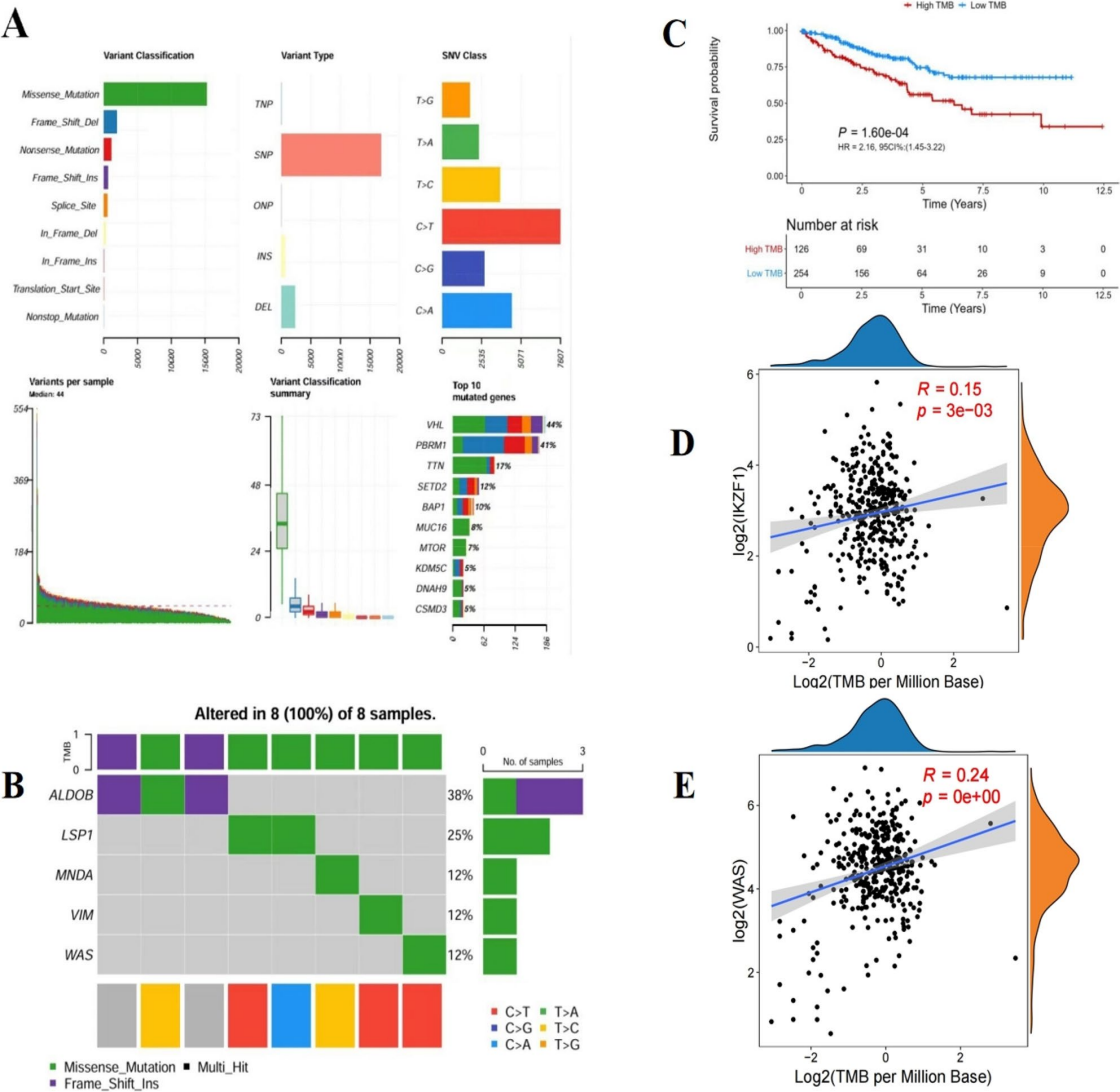
TMB analysis results (**A**)Landscape chart of SNP genes in KIRC samples.**B **Mutation status of co-regulated genes. **C **Survival prognosis K-M curve of high TMB samples. **D**-**E** Correlation of certain genes with TMB levels

To investigate whether the aforementioned co-regulated genes are related to the tumor immune microenvironment, we analyzed the tumor mutational burden (TMB) of RCC samples. As shown in Fig. 3A, the SNP mutation type is predominantly C > T transitions. Among the co-regulated genes, ALDOB exhibited the highest mutation frequency in RCC samples (Fig. 3B), suggesting that it may be involved in the regulation of tumor genomic instability.

Patients were divided into high and low TMB groups based on the median TMB, and survival analysis showed that patients in the high TMB group had a worse prognosis (Fig. 3C). Correlation analysis further indicated that the expression levels of IKZF1 and WAS were significantly positively correlated with TMB (Figs. 3D-E). These results not only provide direct clues for the potential role of ubiquitination-related genes in tumor immunotherapy but also molecularly confirm that the co-regulated ubiquitination-related genes in periodontitis may be associated with tumor mutation accumulation and immune microenvironment remodeling. This provides key molecular markers for exploring the molecular mechanisms of PD as a risk regulatory factor for RCC and opens up cross-research directions for tumor immunotherapy targeting ubiquitination and PD prevention. On this basis, this study further focused on the mutation characteristics of URGs through tumor mutation burden and gene mutation landscape analysis, finding that the ALDOB gene had the highest mutation rate in RCC samples, suggesting that it may be related to the regulation of the tumour immune microenvironment.

### Prognostic value assessment of co-regulated genes

**Fig. 4:**
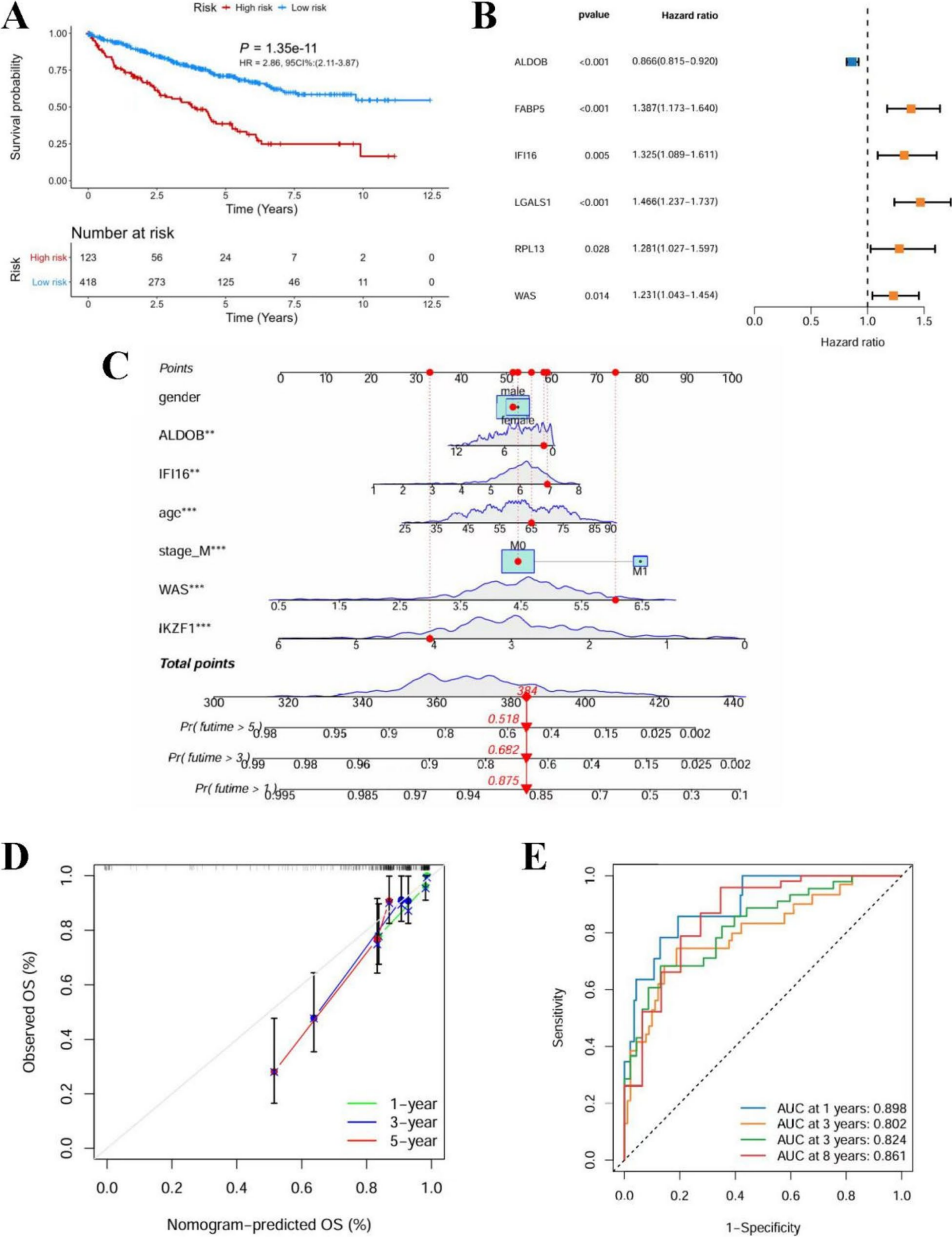
Survival analysis results. **A** Kaplan-Meier survival curves for six URGs significantly associated with overall survival. **B** Forest plot from multivariate Cox regression analysis. **C** Nomogram for predicting survival probability. **D** Calibration curve. **E** Time-dependent ROC curves for 1/3/5/8-year survival predictions

Previous studies have confirmed that the expression differences of the co-regulated ubiquitination-related genes in periodontitis are closely related to the accumulation of tumor mutations and the remodeling of the immune microenvironment in RCC.On this basis, to evaluate the clinical significance of these co-regulated genes, we performed survival analysis on RCC patients to further clarify the association between shared ubiquitination-related genes and prognosis of RCC patients.Kaplan–Meier univariate survival analysis was first conducted. The results showed that among the 10 shared ubiquitination-related genes, the expression levels of six genes (ALDOB, FABP5, IFI16, LGALS1, RPL13 and WAS) were significantly correlated with overall survival (OS) of patients (*P* < 0.05, Fig. 4A).Among them, low expression of ALDOB was associated with poor prognosis, while high expression of FABP5, IFI16, LGALS1, RPL13, and WAS all indicated a poorer prognosis.

To further screen genes with independent prognostic value and eliminate the effects of collinearity among genes and confounding factors (e.g., age, gender, tumor stage), multivariate Cox proportional hazards regression analysis was performed on the six positive genes identified by Kaplan–Meier analysis (Fig. 4B).The nomogram constructed based on these genes and clinical characteristics showed good calibration in predicting 1, 3, and 5-year survival rates (Fig. 4C-E). These results all indicate that these ubiquitination-related genes are not only potential biomarkers for RCC, but may also participate in regulating the disease progression and prognosis of RCC.Finally, four genes with independent prognostic value were identified (ALDOB, IFI16, WAS and IKZF1)(*P* < 0.05). Notably, IKZF1, a ubiquitination-related candidate gene, showed no significant prognostic value in univariate Kaplan–Meier analysis, whereas it exhibited independent prognostic significance after being incorporated into the multivariate regression model.

These findings not only provide potential molecular markers for the accurate prognostic evaluation of RCC but also establish an association between periodontitis and RCC at the survival analysis level, offering critical theoretical and data support for the development of cross-disease diagnosis and treatment strategies based on ubiquitination co-regulated genes.

### Functional enrichment analysis reveals key pathways

**Fig. 5:**
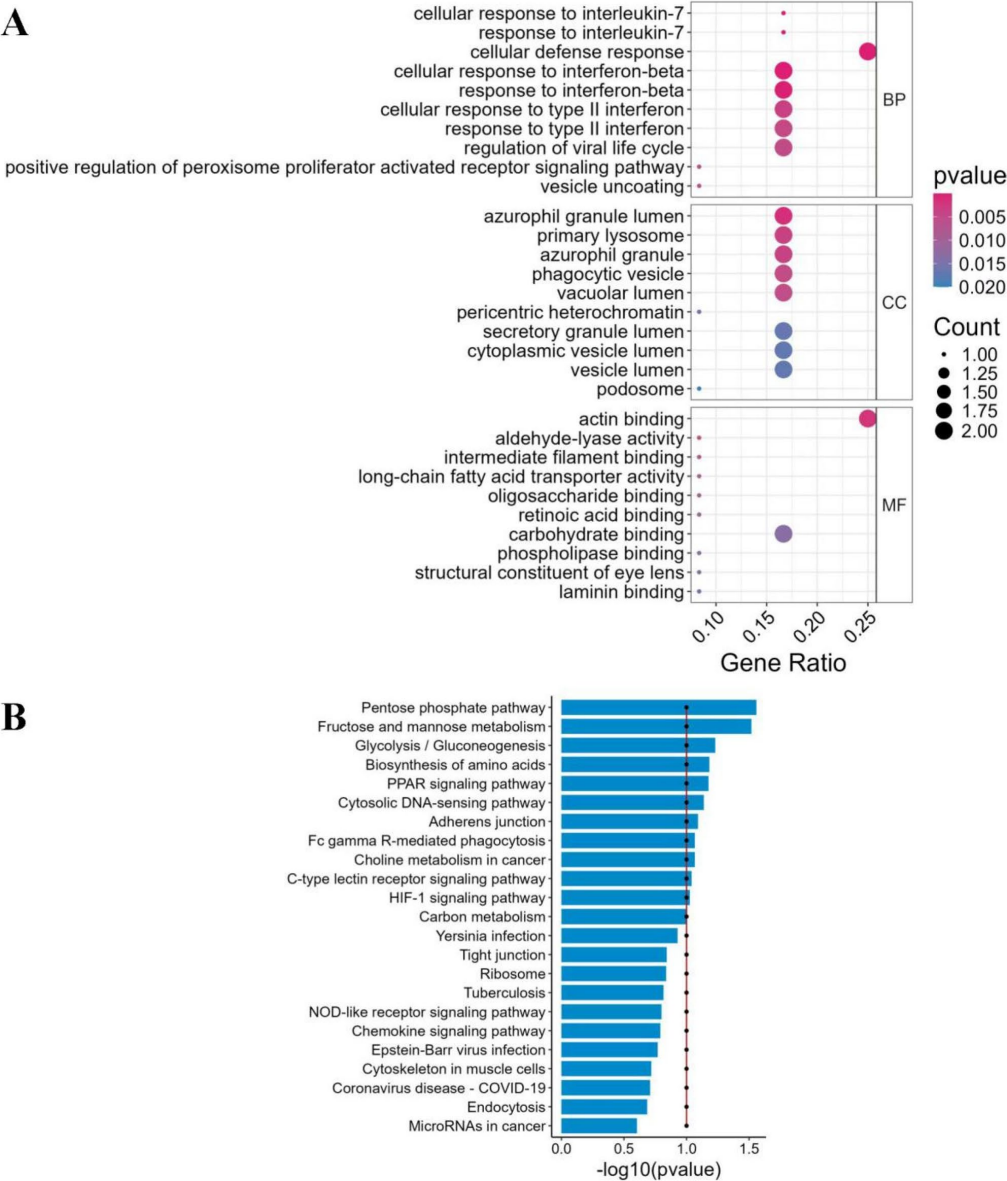
Functional enrichment analysis. **A** GO enrichment analysis. **B** KEGG pathway enrichment analysis

To explore the functional properties and potential signaling mechanisms of the ten ubiquitination-related genes shared by RCC and periodontitis, we performed Gene Ontology (GO) and Kyoto Encyclopedia of Genes and Genomes (KEGG) pathway enrichment analyses.GO enrichment results showed that these genes were mainly enriched in biological processes including mRNA metabolic regulation, translational initiation and cellular response to interferon stimulation (Fig. 5A).KEGG analysis revealed significant enrichment in the p53 signaling pathway, ubiquitin-mediated proteolysis, carbon metabolism and ribosome pathway (Fig. 5B).These enrichment results suggest that these genes may jointly modulate inflammatory and tumorigenic progression via regulating protein stability and metabolic reprogramming, providing a basis for further investigation into the specific roles and molecular mechanisms of these PD–RCC co-regulatory genes.

### Machine learning screening of core diagnostic genes

**Fig. 6:**
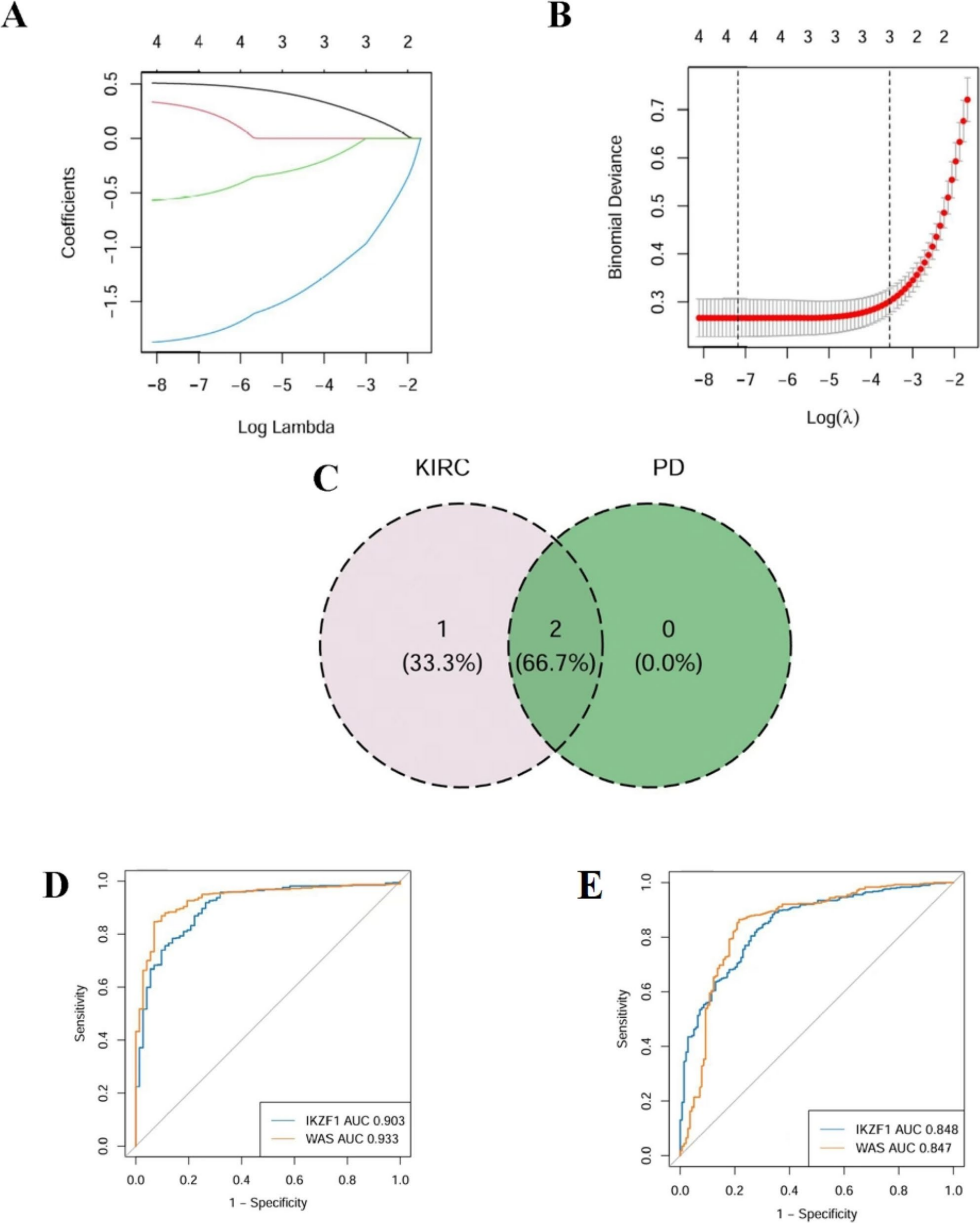
Machine learning for key gene identification. **A**-**B** LASSO regression coefficient paths for KIRC. **C** Venn diagram of key genes coregulated by KIRC and PD. **D**-**E** ROC curves for IKZF1 and WAS in (**D**) RCC and (**E**) periodontitis

First, Kaplan-Meier survival analysis was performed to screen six genes significantly correlated with the prognosis of renal cell carcinoma (RCC) from ten co-regulated genes. Subsequently, multivariate Cox regression analysis further narrowed down the candidates to four genes with independent prognostic values, namely ALDOB, IFI16, WAS and IKZF1. To further identify the core biomarkers, the least absolute shrinkage and selection operator (LASSO) regression algorithm was applied for feature selection among the four candidate genes.LASSO regression selects variables with the greatest contribution to disease status discrimination by penalizing coefficients and shrinking irrelevant variables. Figure 6 A-C illustrates the tuning process of the penalty parameter λ and the variation trajectory of gene coefficients in LASSO regression. At the optimal λ value, the coefficients of ALDOB and IFI16 were shrunk to zero, whereas those of WAS and IKZF1 remained non-zero, indicating that the latter two genes possess the strongest regulatory weights in the comorbidity model of RCC and periodontitis.Finally, IKZF1 and WAS were identified as the core discriminative co-regulatory genes.Meanwhile, ROC curve analysis demonstrated that the AUC values of IKZF1 for distinguishing RCC vs. control and periodontitis vs. control were 0.903 and 0.848, respectively, while the corresponding AUC values of WAS were 0.933 and 0.847 (Fig. 6D-E). These results indicate that both genes exhibit favorable diagnostic performance in the two diseases.

Notably, ALDOB and IFI16 were excluded in LASSO regression. The reasons for their exclusion are systematically elaborated from three aspects as follows: First, the core mechanism of LASSO regression is to shrink gene regression coefficients through the penalty parameter λ, thereby eliminating genes with weak prognostic predictive value, redundant information, or insufficient stability, while prioritizing the retention of genes with more significant contributions. The exclusion of ALDOB and IFI16 is a direct result of this mechanism. Second, although ALDOB and IFI16 were confirmed to have independent prognostic value by multivariate Cox regression, their prognostic effect sizes were lower compared with WAS and IKZF1 (with smaller absolute values of regression coefficients and HR values closer to 1), contributing weakly to the predictive accuracy of the model and showing limited independent predictive ability, thus being prioritized for exclusion. Third, there was potential weak collinearity between ALDOB, IFI16, WAS, and IKZF1, leading to information overlap and model redundancy. The strict penalty mechanism of LASSO regression eliminated such genes to ensure the simplicity, stability, and clinical predictive value of the prognostic model.In addition, ALDOB also had problems such as inconsistent cross-disease expression patterns, weak correlation with the tumor immune microenvironment, and unclear upstream and downstream regulatory pathways, making it unable to be included in the regulatory network analysis. Based on the above comprehensive factors, neither ALDOB nor IFI16 was identified as core ubiquitination-related genes, which further highlights the critical regulatory roles of IKZF1 and WAS in the comorbidity mechanism of renal cell carcinoma (RCC) and periodontitis (PD).

Machine learning analysis not only further confirmed the core roles of the IKZF1 and WAS genes, providing data support for subsequent targeted research, but also suggested that they may synergistically regulate inflammation and tumor progression through the ubiquitination pathway, representing potential therapeutic targets across diseases. Based on the key roles of WAS and IKZF1 in the comorbidity mechanisms of RCC and periodontitis, we selected IKZF1 and WAS for subsequent analysis and experimental validation.

### Regulatory network

**Fig. 7:**
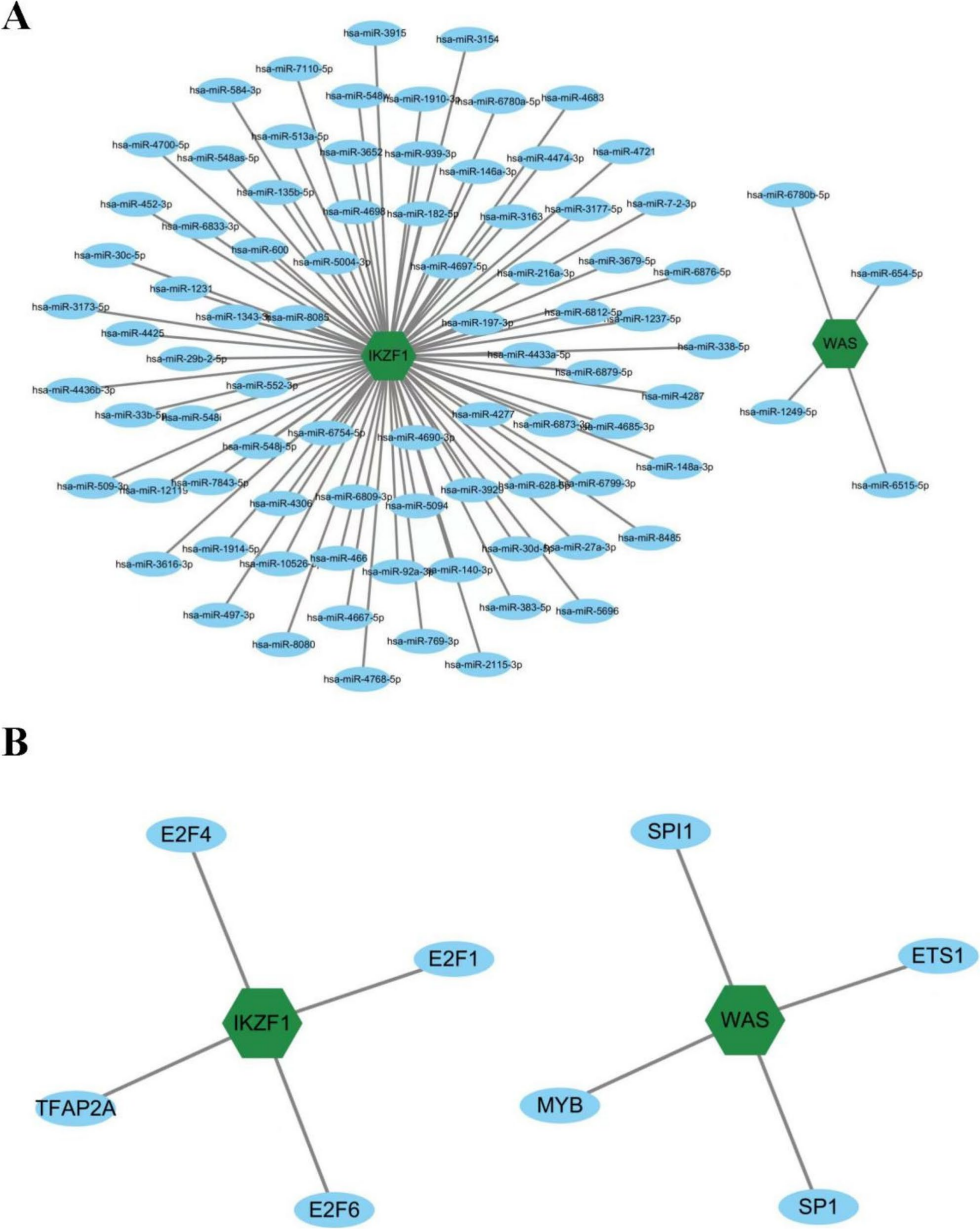
Regulatory network analysis. **A** miRNA-prognostic gene regulatory network. **B** TF-prognostic gene network

Finally, to explore the complexity and diversity of the regulatory processes of key co-regulated genes and gain a deeper understanding of their molecular regulatory mechanisms, the miRDB and miRWalk databases were used to predict miRNAs targeting the prognostic genes. The “VennDiagram” R package was employed to identify the intersection of miRNAs predicted by the two databases. A miRNA-prognostic gene regulatory network was then constructed using Cytoscape software (Fig. 7A). Based on the identified prognostic genes, miRNet was used to predict their corresponding transcription factors (TFs) and obtain information on their interactions. A TF-prognostic gene network was constructed using Cytoscape software (Fig. 7B). Network analysis suggested that the co-regulated genes are not only functionally associated but may also be regulated by a common transcriptional program. Protein-protein interaction (PPI) network analysis provided important clues for understanding the collaborative regulatory mechanisms of these genes.

### Experimental verification of key gene expression

**Fig. 8:**
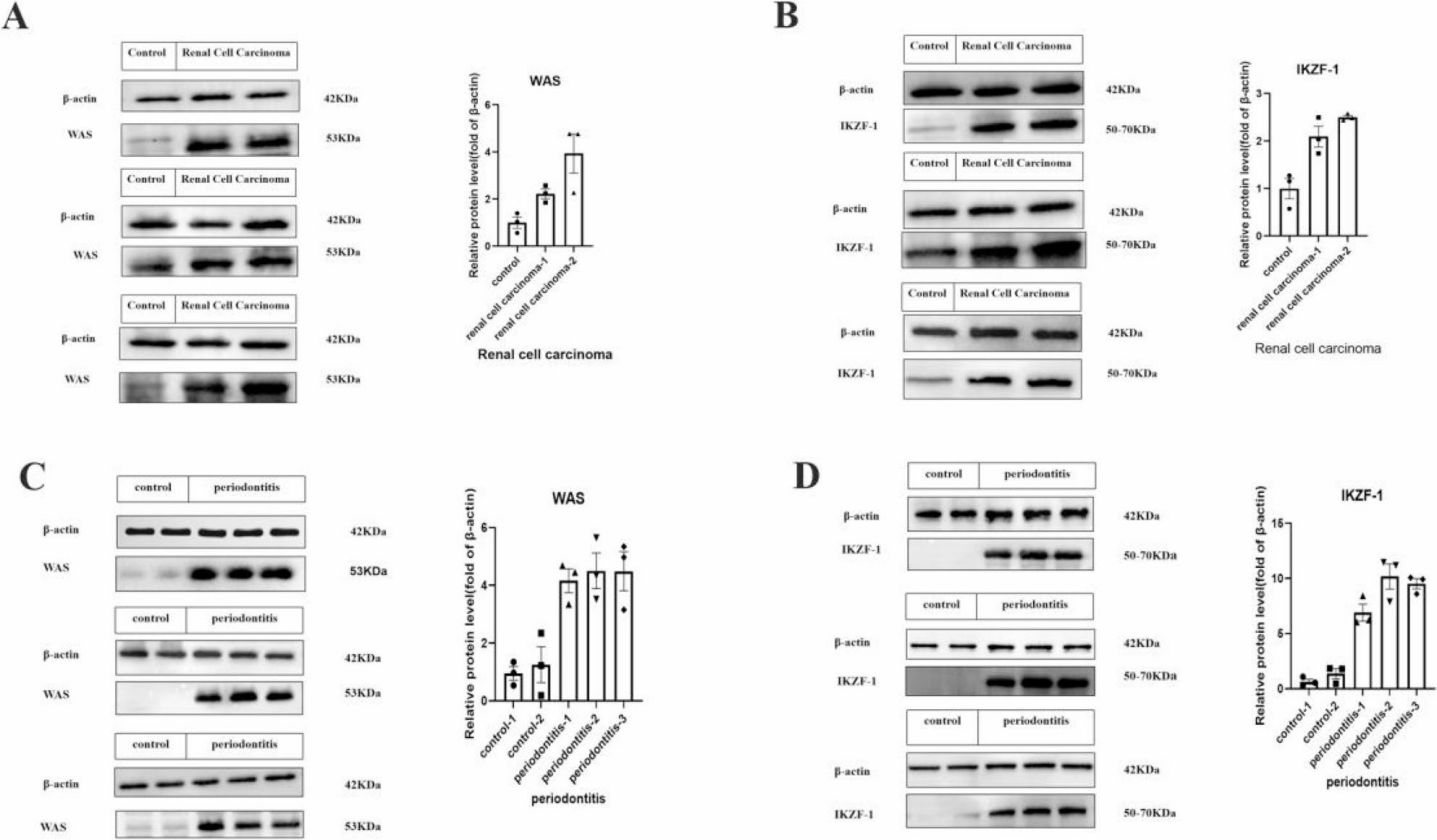
Western blot analysis of IKZF1 and WAS expression. **A**-**B** Protein expression levels in RCC tumor tissues vs. adjacent normal tissues. **C**-**D** Protein expression levels in periodontitis tissues vs. healthy gingival tissues

**Fig. 9:**
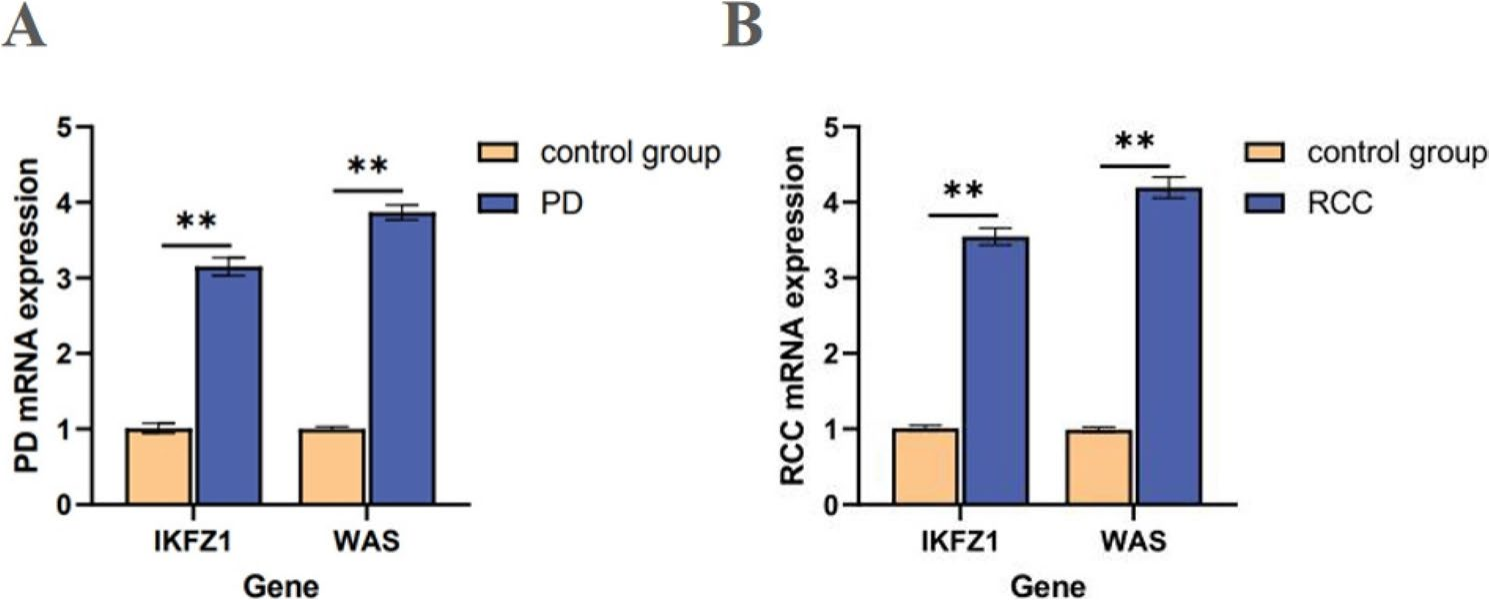
qPCR detection of IKZF1 and WAS gene expression. **A** Compared with the control group, the mRNA expression levels of IKZF1 and WAS were significantly increased in periodontitis (PD) patient samples (P < 0.01). **B** Similarly, the mRNA expression levels of IKZF1 and WAS were significantly upregulated in renal cell carcinoma (RCC) patient samples compared with the control group (P < 0.01)

To validate the results of bioinformatics analysis at the transcriptional and protein levels, we conducted Western blot (WB), quantitative polymerase chain reaction (qPCR), and immunohistochemistry (IHC) assays on the selected core genes IKZF1 and WAS. The results of Western blot and qPCR (Figs. 8 and 9) showed that compared with normal tissues, the protein and mRNA expression levels of IKZF1 and WAS were significantly upregulated in RCC tumor tissues and periodontitis lesions (*p* < 0.001).

**Fig. 10:**
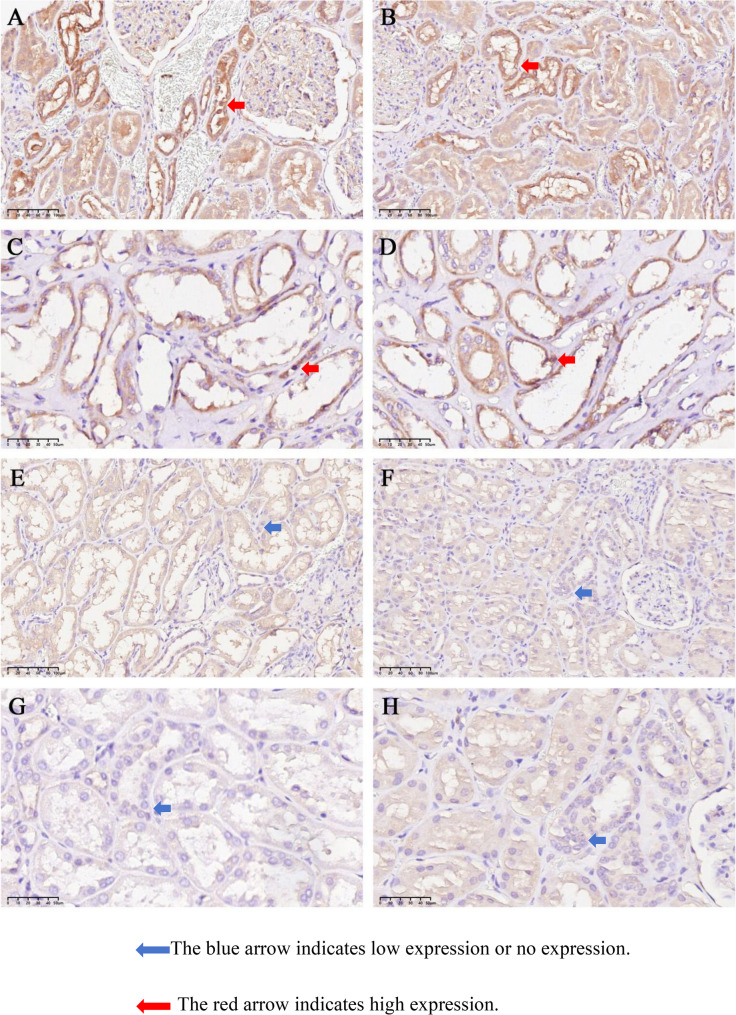
IHC validation of WAS protein expression in RCC. Figure legend: Representative images showing high WAS expression in renal cancer tissues (**A**-**D**) and low expression in adjacent normal tissues (**E**-**H**). Magnification: 20x (A, B, E, F); 40x (C, D, G, H)

**Fig. 11:**
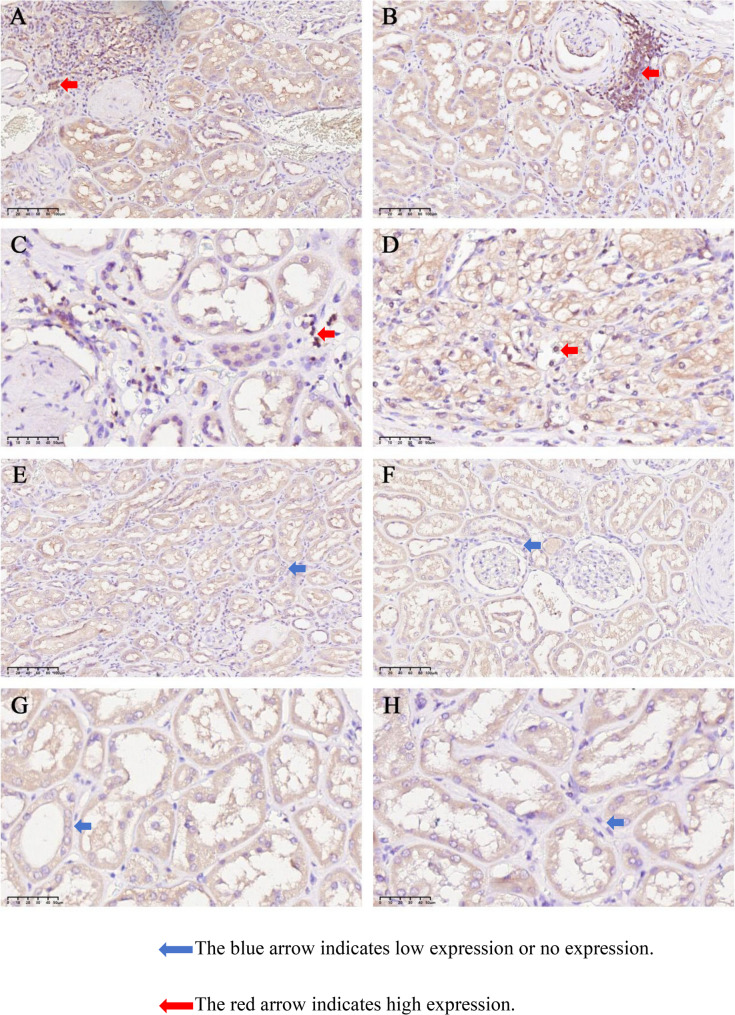
IHC validation of IKZF1 protein expression in RCC. Figure legend: Representative images showing high IKZF1 expression in renal cancer tissues (**A**-**D**) and low expression in adjacent normal tissues (**E**-**H**). Magnification: 20x (A, B, E, F); 40x (C, D, G, H)

**Fig. 12:**
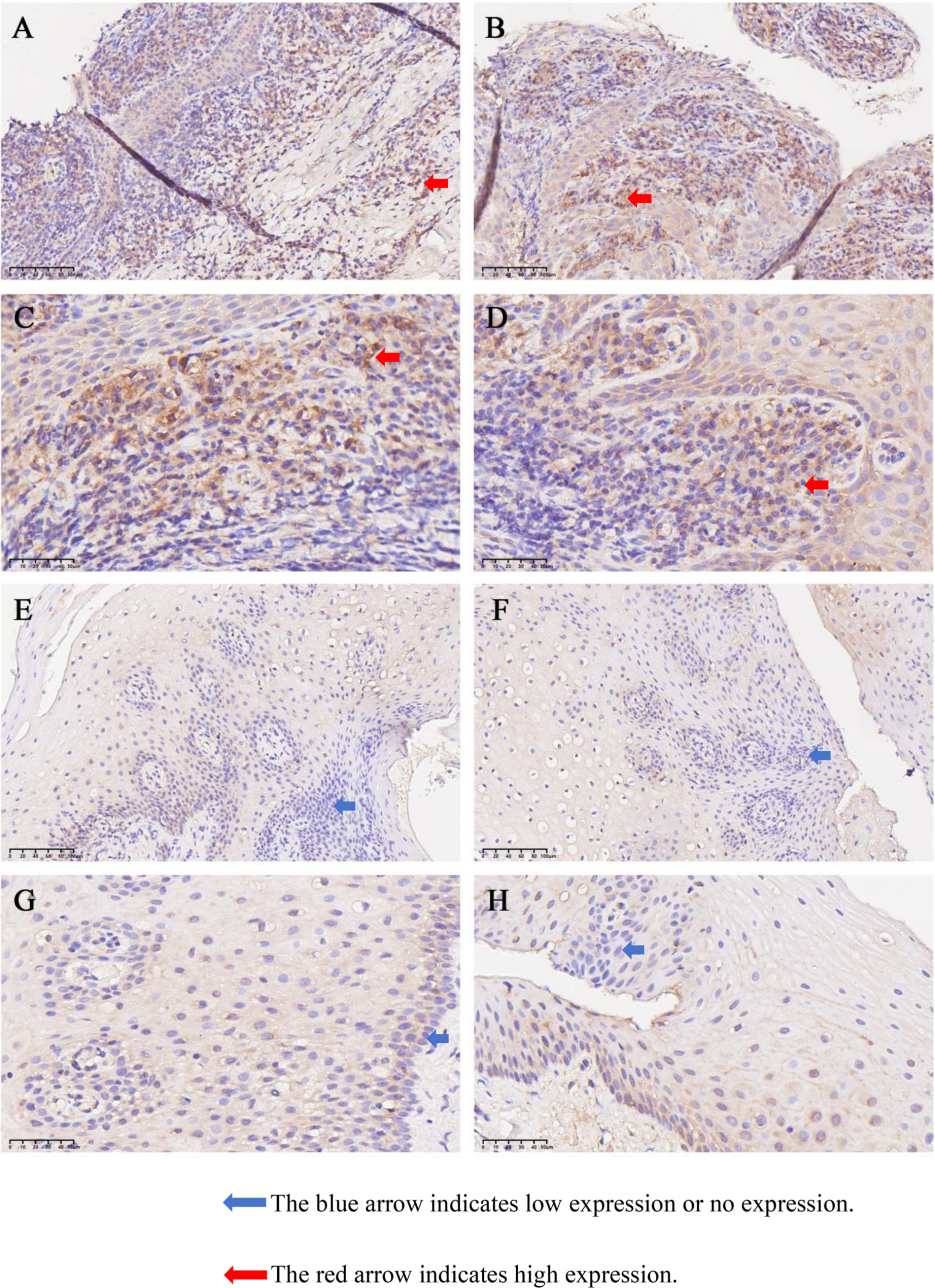
IHC validation of WAS protein expression in periodontitis. Figure legend: Representative images showing high WAS expression in periodontal inflammatory tissues (**A**-**D**) and low expression in healthy periodontal tissues (**E**-**H**). Magnification: 20x (A, B, E, F); 40x (C, D, G, H)

**Fig. 13:**
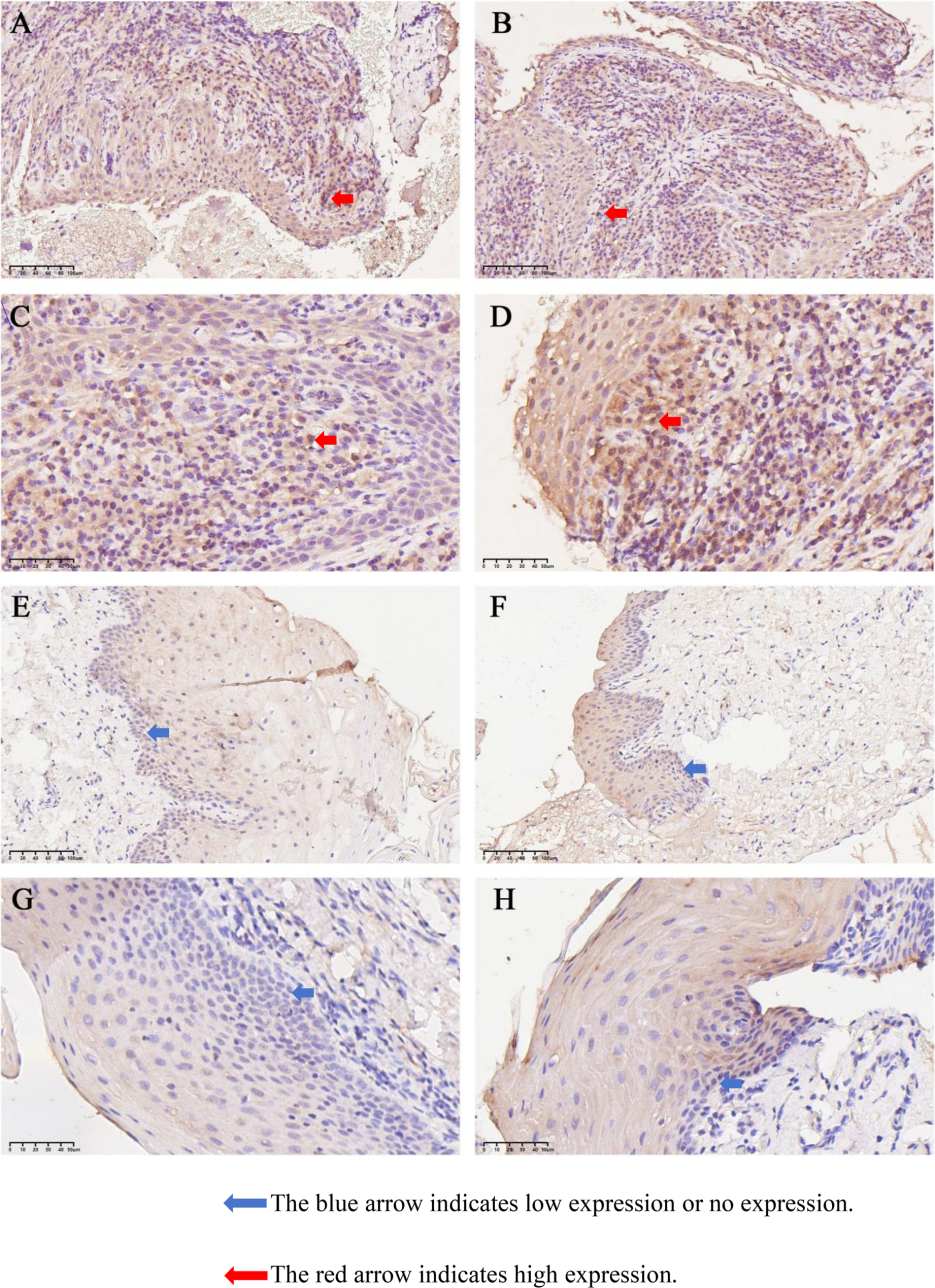
IHC validation of IKZF1 protein expression in periodontitis. Figure legend: Representative images showing high IKZF1 expression in periodontal inflammatory tissues (**A**-**D**) and low expression in healthy periodontal tissues (**E**-**H**). Magnification: 20x (A, B, E, F); 40x (C, D, G, H)

The IHC results further confirmed the differences in protein expression levels. In RCC, the WAS protein was highly expressed in tumor tissues, while its expression was extremely low in adjacent normal tissues (Fig. 10). Similarly, the IKZF1 protein was also significantly overexpressed in tumor tissues (Fig. 11). In periodontitis tissues, the expression levels of WAS and IKZF1 in inflammatory lesion areas were significantly higher than those in healthy periodontal tissues (Figs. 12 and 13). Our experimental results consistently confirmed the common high expression of IKZF1 and WAS in RCC and periodontitis, supporting their role as key molecules linking the two diseases.

## Discussion

The research findings indicate that, on one hand, periodontal disease shares certain common risk factors with renal cell carcinoma [17]; on the other hand, the persistent and low-grade inflammatory immune microenvironment of periodontitis can activate multiple inflammatory pathways, induce imbalance in the oxidation-antioxidation system, and promote abnormal cell proliferation through various pathways, providing a potential pathological microenvironment suitable for tumor growth [8]. Notably, the pathogenesis of RCC is closely associated with core pathological mechanisms, including inflammatory microenvironment remodeling, dysregulated cell cycle regulation, escape from apoptosis, and abnormal invasion and metastasis capabilities [18, 19].These observations suggest potential molecular associations and underlying synergistic effects between RCC and periodontitis (PD) [20, 21].

### The cross-regulatory role of ubiquitination-related genes in renal cell carcinoma and periodontitis

Through systematic transcriptomic analysis and multi-level experimental validation, this study for the first time revealed the overlapping expression characteristics of ubiquitination-related genes in renal cell carcinoma (RCC) and periodontitis, providing experimental evidence for the preliminary exploration of the intrinsic molecular association between the two diseases and their potential common pathogenic mechanisms.During the study,10 ubiquitination-related genes were identified, among which 6 genes (IKZF1, LGALS1, LSP1, MNDA, VIM, and WAS) were significantly upregulated in both diseases. These 6 genes were closely associated with the survival prognosis of RCC patients, suggesting that they may serve as common expression markers for the two diseases and be involved in the progression of both conditions. Meanwhile, these findings imply that ubiquitination may be a potential key link in the development and progression of chronic inflammation and cancer.

Among the 10 ubiquitination-related genes identified in this study, IKZF1 and WAS exhibited the most prominent overlapping expression characteristics in the two diseases, suggesting they may be potential key molecules involved in the pathological processes of both conditions. As a zinc finger transcription factor of the IKAROS family [22], IKZF1 can regulate immune cell differentiation, inflammatory responses, and activate related signaling pathways such as STAT3 [23–28]. Its consistently high expression in both periodontitis (PD) and renal cell carcinoma (RCC) suggests it may be associated with the inflammatory and malignant phenotypes of these diseases. Meanwhile, existing studies have shown that the abnormal expression of IKZF1 is related to the occurrence, invasion and prognosis of various cancers (such as leukemia) [29–31]. As a key regulatory factor in cytoskeleton remodeling, the consistent high expression of WAS in both diseases suggests that it may be involved in the pathological progression of the two conditions [32, 33]. In PD, its overexpression may accelerate the degradation of periodontal supporting tissues by enhancing the cytoskeleton remodeling ability of periodontal ligament cells and immune cells, as well as regulating MMPs [34–37]. In RCC, WAS protein may bind to CDC42 to activate the WAVE2-Arp2/3 signaling pathway and PI3K/Akt signaling pathway, thereby regulating downstream target genes such as FoxO1 and enhancing the invasion, migration and proliferation of tumor cells [38–40].

Furthermore, this study also found that although LGALS1 and VIM are not typical ubiquitination-related genes, as known cancer-related molecules, their consistent upregulation further suggests that ubiquitination-related genes may exhibit a synergistic expression pattern in the two diseases.Overall, the 6 co-upregulated ubiquitination-related genes (URGs) identified in this study may not function independently; instead, they may form a synergistic molecular network through ubiquitination-mediated protein degradation and signaling pathway regulation, collectively promoting the pathological progression of RCC and periodontitis (PD).​Notably, although ALDOB appeared repeatedly in the preliminary screening, it was not ultimately identified as a core ubiquitination-related gene, mainly based on the following considerations: First, ALDOB exhibited different expression patterns between periodontitis and RCC, lacking the consistency necessary for the cross-disease comorbidity mechanism. Second, it had a weak association with the tumor immune microenvironment—unlike IKZF1 and WAS, which were significantly positively correlated with tumor mutational burden (TMB) and directly involved in immune remodeling, ALDOB only showed a relatively high gene mutation rate and could hardly participate in the “inflammation-immunity-tumor” cross pathway. In addition, LASSO regression showed that its regulatory weight was significantly lower than that of IKZF1 and WAS. More importantly, the upstream and downstream regulatory molecules of ALDOB remain unclear, and it could not be included in the Cytoscape regulatory network analysis due to the lack of clear support from targeted miRNA and transcription factor pathways, thus failing to be positioned as a core regulatory gene.This exclusion process further highlights the critical roles of IKZF1 and WAS in the comorbidity mechanism of RCC and PD.

Functional enrichment analysis showed that the screened ubiquitination-related genes were significantly enriched in the p53 signaling pathway and the ubiquitin-mediated protein degradation pathway, suggesting that these pathways may be associated with the expression correlation characteristics of the two diseases. The high expression of core genes may affect the activity and stability of p53 by interfering with the balance of p53 ubiquitination modification, which is speculated to impair its tumor suppressor function in RCC and exacerbate periodontal tissue damage in periodontitis (PD). However, direct mechanistic verification is still lacking for the above associations. In addition, the core genes were also indirectly enriched in inflammation- and tumor-related pathways such as NF-κB, suggesting that they may be involved in activating the NF-κB pathway by regulating the ubiquitination degradation of NF-κB-related proteins, thereby participating in the local inflammation of periodontitis and the progression of RCC. These pathways may be the potential molecular pathways underlying the expression correlation between the two diseases, providing theoretical clues for the subsequent exploration of potential therapeutic strategies related to “inflammation-immunity-tumor”.

### Innovation and limitations of research

The innovation of this study lies in the first systematic analysis of the overlapping expression characteristics of ubiquitination-related genes in RCC and periodontitis from the perspective of comorbidity, and the experimental verification and identification of core regulatory genes. In addition, the findings of this study also provide indirect clues for the “inflammation-induced cancer” hypothesis—systemic inflammation caused by periodontitis may be associated with abnormal expression of ubiquitination-related genes, which in turn may lay a potential pathological foundation for the occurrence and development of RCC. These results not only enrich the research on the molecular association between chronic inflammation and tumorigenesis, but also provide a theoretical basis and experimental support for the cross-disease research of RCC and periodontitis, and offer new ideas for the clinical diagnosis and treatment of related diseases.

However, this study still has certain limitations. Firstly, the data mainly come from public databases, and the inclusion criteria for samples in the public databases are not completely unified. Even with strict sample screening, it is still impossible to completely eliminate the interference of confounding factors on the analysis results of the genedisease association; at the same time, although the data have been batchcorrected and standardized, there are still inherent differences in the sample detection platforms and experimental operation procedures among different databases, and the disease subtypes, clinical stages, etc. of the samples are heterogeneous, which may all affect the universality of the research results. Secondly, the sample size collected during the experimental validation phase was insufficient, and selection bias may exist. This issue must be acknowledged, as the limited sample size undermines the generalizability of the findings and may introduce bias.Thirdly, The periodontitis group included a mixture of chronic and aggressive periodontitis subtypes, while the control group consisted of healthy gingival tissues obtained from patients undergoing other dental procedures. Such non-independent source of controls may introduce selection bias.Key demographic and clinical characteristics, including age, sex, smoking status, and disease severity, were not adequately compared between groups; thus, unbalanced confounding factors may interfere with the interpretation of the results. Therefore, the validation findings should be regarded as exploratory. Future studies with larger sample sizes, strictly matched case–control cohorts, and comprehensive adjustment for confounders are warranted to replicate our results.Fourthly, the depth of mechanistic investigation is insufficient, which represents a major limitation of this study. Although we validated the differential expression of key genes such as WAS and IKZF1 at both the protein and transcriptional levels using WB, qPCR, and IHC, we did not conduct targeted experimental investigations into the functional roles or ubiquitination-related mechanisms of these genes. Experiments such as ubiquitination assays or pathway modulation were not performed, leaving a lack of direct experimental evidence. Furthermore, this study only provided a preliminary exploration of gene expression levels and cellular functions, without in-depth investigation of post-translational modifications or specific upstream and downstream regulatory pathways. The related molecular functional mechanisms remain incompletely understood, and the functional roles and regulatory relationships of these genes have not been further validated through cellular or animal experiments.Fifth, differential expression analysis in the present study was performed without multiple testing correction, using only a *p* < 0.01 threshold and fold-change cutoff for gene screening. Although this approach helps reduce the risk of false negatives in preliminary screening, it may introduce a proportion of false-positive genes into downstream analyses.Notably, the key genes identified in this study (e.g., IKZF1 and WAS) were not determined solely based on differential expression. Instead, they were subjected to multiple independent rounds of filtering, including survival analysis, LASSO regression, and experimental validation using clinical specimens. Thus, the robustness and reliability of the main conclusions were not significantly compromised.In future investigations, we will apply the more stringent Benjamini-Hochberg FDR correction for differential expression analysis to further improve the rigor, reproducibility, and reliability of our results.

Therefore, the current findings should be considered as preliminary experimental results.Future studies should focus on expanding the clinical sample size, standardizing sample selection criteria, clarifying sample subtype classification, and adequately comparing sample characteristics between groups to minimize selection bias. In parallel, cellular and animal experiments, along with ubiquitination-specific assays and pathway modulation experiments, should be conducted to systematically elucidate the regulatory functions, molecular mechanisms, and translational value of WAS, IKZF1, and other genes in RCC and PD, and to further validate the associations between these genes and the two diseases, thereby addressing the current research gaps.

### Clinical translation potential

From a clinical perspective, the ubiquitination-related genes screened in this study have the potential to serve as biomarkers or therapeutic targets. For example, the expression levels of IKZF1 and WAS may be used to predict the prognosis of RCC patients or the severity of PD. Furthermore, ubiquitination inhibitors or agonists targeting these genes may become new strategies for cross-disease treatment. Especially in the context of precision medicine, drugs targeting the ubiquitination pathway (such as proteasome inhibitors) have been used in the treatment of various cancers. This study provides a scientific basis for expanding their indications.

### Future research directions

Future research should focus on the following specific directions:1)Conduct functional assays, animal models, and ubiquitination‑specific experiments to clarify the potential causal relationships between ubiquitination‑related genes and the pathological progression of RCC and PD, and to further investigate their upstream and downstream regulatory pathways.2)Expand clinical sample sizes and carry out multicenter clinical studies to validate the reliability of core genes as potential biomarkers.3)Develop small‑molecule inhibitors or gene therapies targeting the core genes and explore their potential clinical translational value.4)Investigate the crosstalk between ubiquitination and other post-translational modifications (e.g., phosphorylation, acetylation) to refine the regulatory networks associated with gene expression.5)Further explore the potential mechanisms by which ubiquitination‑related genes influence the progression of RCC and PD through modulation of the immune microenvironment, and clarify the molecular association characteristics between the two diseases.

In summary, through multi-omics analysis and experimental validation, this study revealed the consistent expression characteristics of ubiquitination-related genes in RCC and periodontitis (PD), providing important clues for understanding the potential common mechanisms of the two diseases and developing novel therapeutic strategies. These findings not only enrich the relevant theories regarding the expression characteristics of ubiquitination in diseases, but also provide a preliminary model for interdisciplinary research.

## Conclusion

This research, which was carried out, has made known the mechanism of cross - regulation of genes related to ubiquitination in Renal Cell Carcinoma (RCC) and Parkinson’s Disease (PD) by means of conducting a transcriptomic analysis. These genes do not just offer brand - new understandings regarding the molecular mechanisms of the two diseases, but in addition, they offer potential things that can serve as biomarkers and targets for treatment for medicine with precision in the future and the development of drugs.

## The items of materials and the ways of methods

### The action of data acquisition and the process of preprocessing

Use the R package TCGA biolinks to download the TCGA-KIRC data set (November 2024), 541 tumors and 72 controls, and all tumor samples have survival data. Use the R package GEOquery to download GEO periodontitis (PD) data sets: GSE10334 (GPL570 platform, disease group 183, control group 64), GSE16134 (GPL570 platform, disease group 241, control group 69), and GSE106090 (GPL21827 platform, disease group 6, control group 6). The three PD data sets were then merged. First, the normalizeBetweenArrays function of the R package limma was used to standardize between samples, and then the ComBat function was used to remove the batch effect between different data sets. Data Source: RNA-seq data from the TCGA database (541 tumor samples and 72 control samples) and gene expression data from the GEO database (GSE10334, GSE16134, and GSE106090). Data Preprocessing: Sample normalization was performed using the limma package, and batch effects were removed using the Combat function.

### Differential expression analysis

The differential genes between the disease group and the control group were analyzed using the limma package (version 3.60.4). The threshold for the KIRC dataset was pvalue < 0.01 and |log2FC| > 1; for the PD dataset, it was pvalue < 0.01 and |log2FC| > 0.5. A total of 3,604 differentially expressed genes (DEGs) were obtained from the TCGA-KIRC dataset, including 1,621 upregulated genes and 1,983 downregulated genes. Differential expression analysis was performed using the limma package, and the differentially expressed genes (DEGs) in RCC and PD were selected. The selection criteria for RCC were pvalue < 0.01 and |log2FC| >1, and for PD, they were pvalue < 0.01 and |log2FC| >0.

### Screening of ubiquitination-related genes

From the literature with PMID: 39,367,086, 332 ubiquitination-related genes were collected. By taking the intersection with the DEGs of KIRC and PD, a total of 10 genes were obtained, namely ALDOB, FABP5, IFI16, IKZF1, LGALS1, LSP1, MNDA, RPL13, VIM, and WAS. These genes were labeled as candidate genes for co-regulation in KIRC-PD. From the literature, 332 ubiquitination-related genes were collected and intersected with the DEGs of RCC and PD to obtain 10 co-regulated genes.

### Analysis of Tumor Mutation Burden (TMB)

To explore whether these co-regulated genes are related to the tumor immune microenvironment, we performed tumor mutation load analysis. The TMB analysis process mainly includes three key steps: data preparation, mutation detection, and TMB calculation. First, BAM/FASTQ files of tumor and normal samples, reference genome and target region files are required. Then, use tools such as GATK Mutect2 to detect somatic mutations, generate VCF files, filter germline mutations by matching normal samples, and eliminate low-quality mutations. Finally, calculate the TMB value. The formula is the number of effective mutations divided by the size of the target area (unit: Mb). For example, WES is calculated as approximately 30-50 Mb, and Panel is adjusted according to the actual size (such as 1.5 Mb). The results are expressed as the number of mutations/Mb and are used to evaluate the potential efficacy of immunotherapy. The entire process requires strict quality control to ensure the accuracy of mutation annotation and region size.

### Survival analysis

Survival analysis of the expression of each gene found that the expression of some genes was significantly related to survival prognosis. A high expression survival number indicates that the gene is a protective gene, and vice versa. A Cox proportional hazards model was subsequently applied to assess the prognostic significance of ubiquitination-related genes in RCC.

### Functional enrichment analysis

GO and KEGG pathway enrichment analysis was conducted using the `clusterProfiler` package to explore the biological functions of ubiquitination-related genes.

### Machine learning

Key co-regulated genes were further screened using Lasso machine learning using 4 genes (ALDOB, IFI16, WAS, and IKZF1) that were significant in multiple regression.

### Protein-protein interaction network construction

The PPI network was constructed using Cytoscape software to analyze the interaction relationships of ubiquitination-related genes.

### Regulatory network

Finally, in order to explore the complexity and diversity of the expression regulation process of key co-regulated genes and thereby gain a deeper understanding of the molecular regulatory mechanism of gene expression, miRDB and miRWalk databases were used to predict miRNAs targeting prognostic genes. The “VennDiagram” R package was used to take the intersection of the predicted miRNAs in the databases. The miRNAprognostic gene regulatory network was constructed using Cytoscape software.

### Research subjects

All clinical samples and the experimental protocols of this study were strictly reviewed and approved by the Ethics Committee of Shanxi Provincial People’s Hospital (Ethical Approval Number: No. 335). All research subjects signed written informed consent forms. The entire research process strictly followed the Helsinki Declaration and relevant medical ethics guidelines. Sample collection, preservation, and processing all complied with clinical biological sample management standards.

All research subjects were selected from inpatients and outpatients of Shanxi Provincial People’s Hospital during the period from January 2024 to December 2025. Samples of renal cell carcinoma and adjacent normal tissues were collected from newly diagnosed patients who underwent radical or partial nephrectomy. All case samples were confirmed as clear cell renal cell carcinoma by pathologists according to the WHO (2022) classification criteria, and the AJCC 8th edition stage and WHO/ISUP grade were recorded. The inclusion criteria for tissue sampling before collection were: no prior anti-tumor treatment such as radiotherapy,

chemotherapy, or targeted therapy, and complete clinical data. Patients with extrarenal metastasis, kidney organic diseases, or non-clear cell cancer were excluded. A total of 20 tissue samples were collected, divided into 10 cases of the cancer group and 10 cases of the cancer adjacent group (normal kidney tissue≥5 cm away from the tumor focus, confirmed by the pathology department without tumor cell infiltration). Both groups of samples were quickly dissected during surgery, immediately frozen in liquid nitrogen, and transferred to a -80 °C ultra-low temperature refrigerator for RNA extraction and protein extraction. Some tissue blocks (approximately1.0 cm×1.0 cm×0.5 cm) were fixed with 10% neutral formalin for 24–48 h, then routinely dehydrated and wax-embedded for immunohistochemistry (IHC) detection. This study intends to collect gingival tissue specimens from three groups of people: the chronic periodontitis group, the aggressive periodontitis group, and the periodontal health control group. General inclusion criteria (applicable to all subjects): age ≥ 18 years; good overall health, no systemic diseases; at least 18 natural teeth; good patient compliance, able to cooperate with clinical examinations and sample collection; no periodontal-related treatment in the past two years; no use of orthodontic appliances. Specific inclusion criteria for each group:①Chronic periodontitis group: age > 35 years; meeting the diagnostic criteria for generalized chronic periodontitis in the 1999 American Periodontal Disease Society classification; more than 30% of the sites in the entire mouth have clinical attachment loss (CAL)≥3 mm; sites with probing depth≥5 mm; positive probing on bleeding (BOP).②Aggressive periodontitis group: age between 18 and 35 years; meeting the diagnostic criteria for generalized aggressive periodontitis in the 1999 American Periodontal Disease Society classification; at least 6 permanent teeth (incisors and first molars) have at least one site with CAL≥5 mm and probing depth≥5 mm; at least 3 other permanent teeth have at least one site with probing depth≥5 mm and CAL≥5 mm; positive on BOP.③Periodontal health control group: all sites have probing depth≤3 mm; no clinical attachment loss (CAL); no obvious alveolar bone resorption on imaging; bleeding index (BI) ≤ 1; samples were from healthy gingival tissues to be subjected to crown lengthening surgery, orthodontic extraction, or impacted tooth extraction. A total of 20 gingival tissue samples were collected, divided into 10 cases of the periodontal inflammation group and 10 cases of the normal group. The sample collection and preservation method was the same as that of RCC samples.

### Experimental verification

Western blot and qPCR analyses were conducted to verify the expression differences of key genes IKZF1 and WAS. Immunohistochemistry (IHC) verification: Use IHC technology to detect the protein expression levels of key genes in RCC and PD tissues. All operations were approved by the ethics committee, and informed consent was obtained from patients. All tissue specimens were used only for this experimental study and were strictly prohibited from being used for any commercial purposes or non-research purposes. All the information related to the specimens has been anonymized, and the personal identity information of the donors has been concealed. During the research process, the tissue specimens were used strictly in accordance with the ethical approval requirements and the experimental plan, and the number of specimens was reasonably controlled to avoid waste.

## Supplementary Information

Supplementary Material 1

## Data Availability

All data generated or analysed during this study are included in this published article and its supplementary information files.

## Abbreviations

PD: Periodontitis
RCC: Renal cell carcinoma
PCA: Principal Component Analysis
URGs: Ubiquitination-related genes
TMB: Tumor mutational burden
GO: Gene Ontology
KEGG: Kyoto Encyclopedia of Genes and Genomes
TF: Transcription factors
IHC: Immunohistochemistry
qPCR: Quantitative polymerase chain reaction
DEGs: Differentially expressed genes
CAL: Clinical attachment loss
BOP: Bleeding on probing
BI: Bleeding index
