# Supplementary material for: Overlapping expression characteristics of ubiquitination-related genes in periodontitis and renal cell carcinoma: transcriptomic analysis and experimental validation
Source: BMC Oral Health. 2026 May 19;26:1340. doi: 10.1186/s12903-026-08574-2 (PMC13397633; doi:10.1186/s12903-026-08574-2)
Supplement: Supplementary file 1 — Supplementary Material 1 [file 12903_2026_8574_MOESM1_ESM.docx]

**IKZF-1:50-70KDa**

**WAS:53KDa**

**β-actin:42KDa**

**①Periodontitis+IKZF-1(Target protein):**

**Group 1**


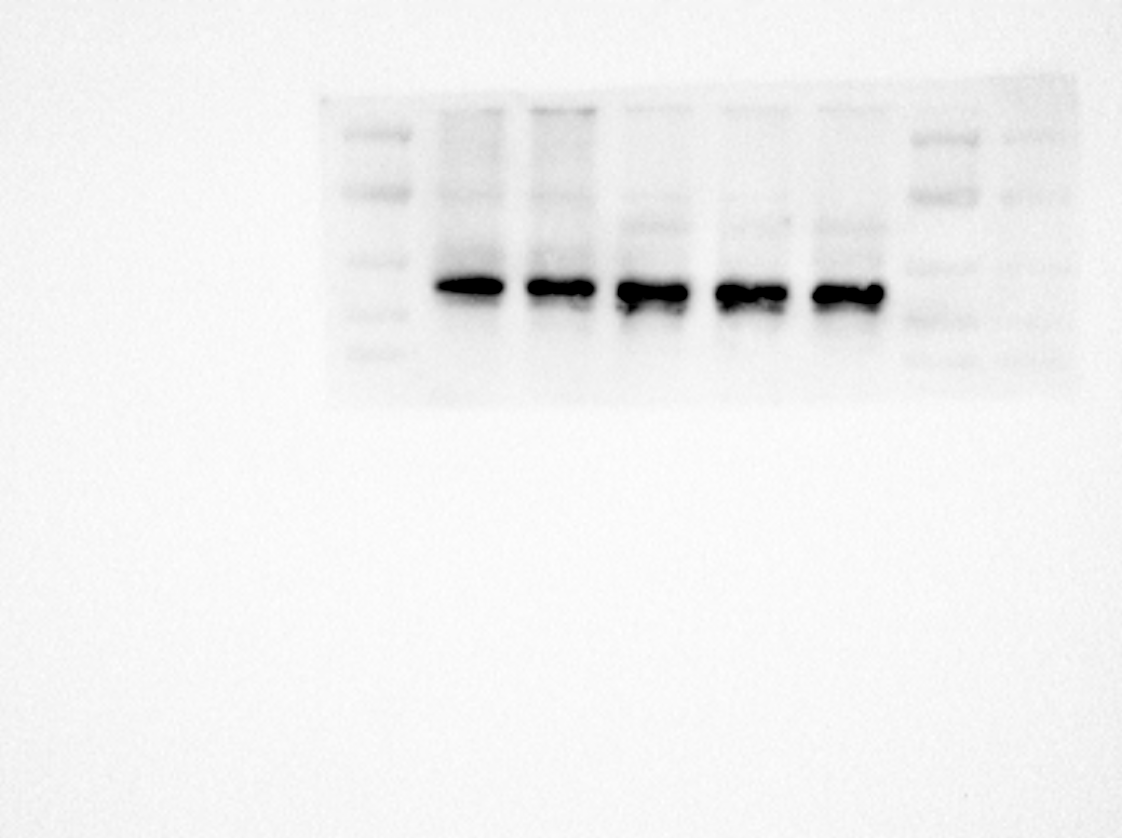

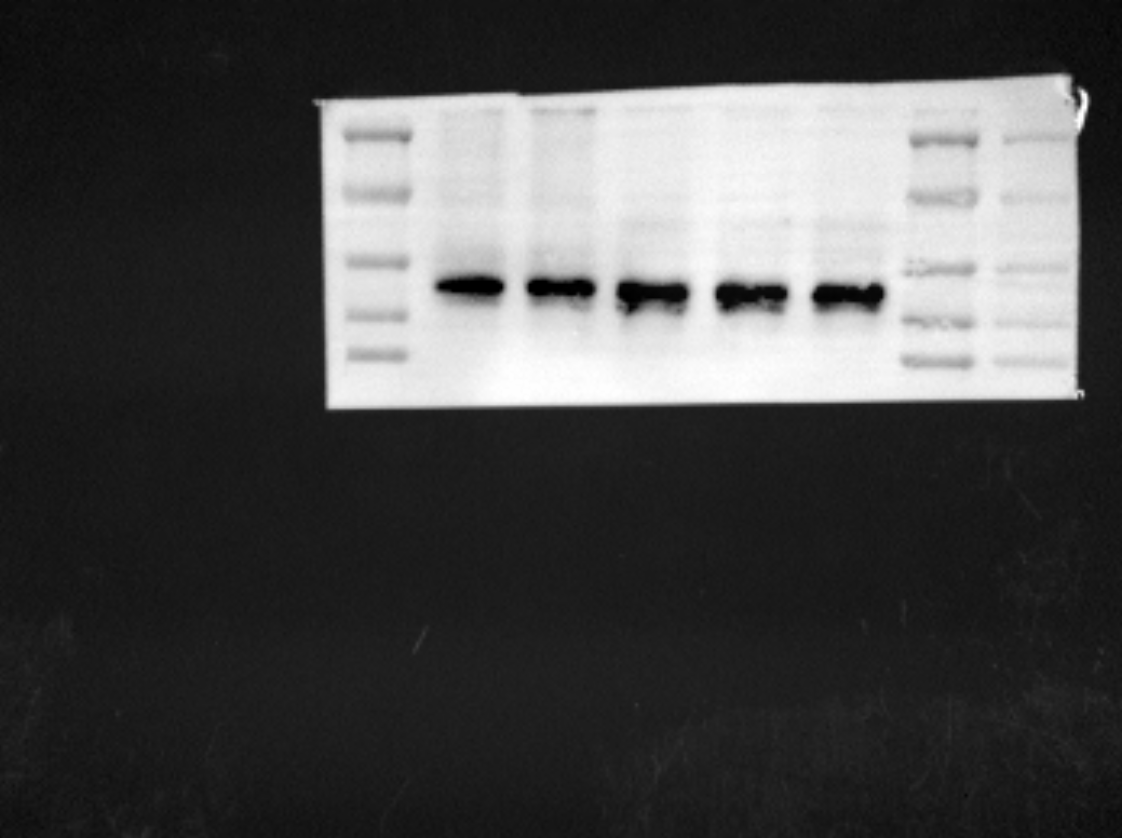

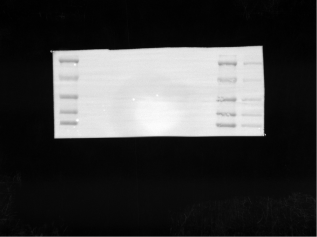


β-actin β-actin +marker marker

**
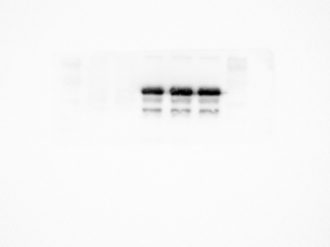

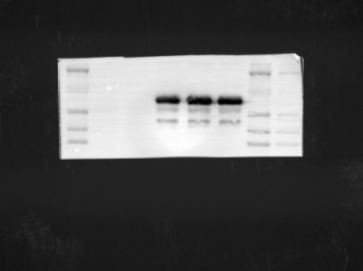
**

IKZF-1 IKZF-1+marker

**Group 2**


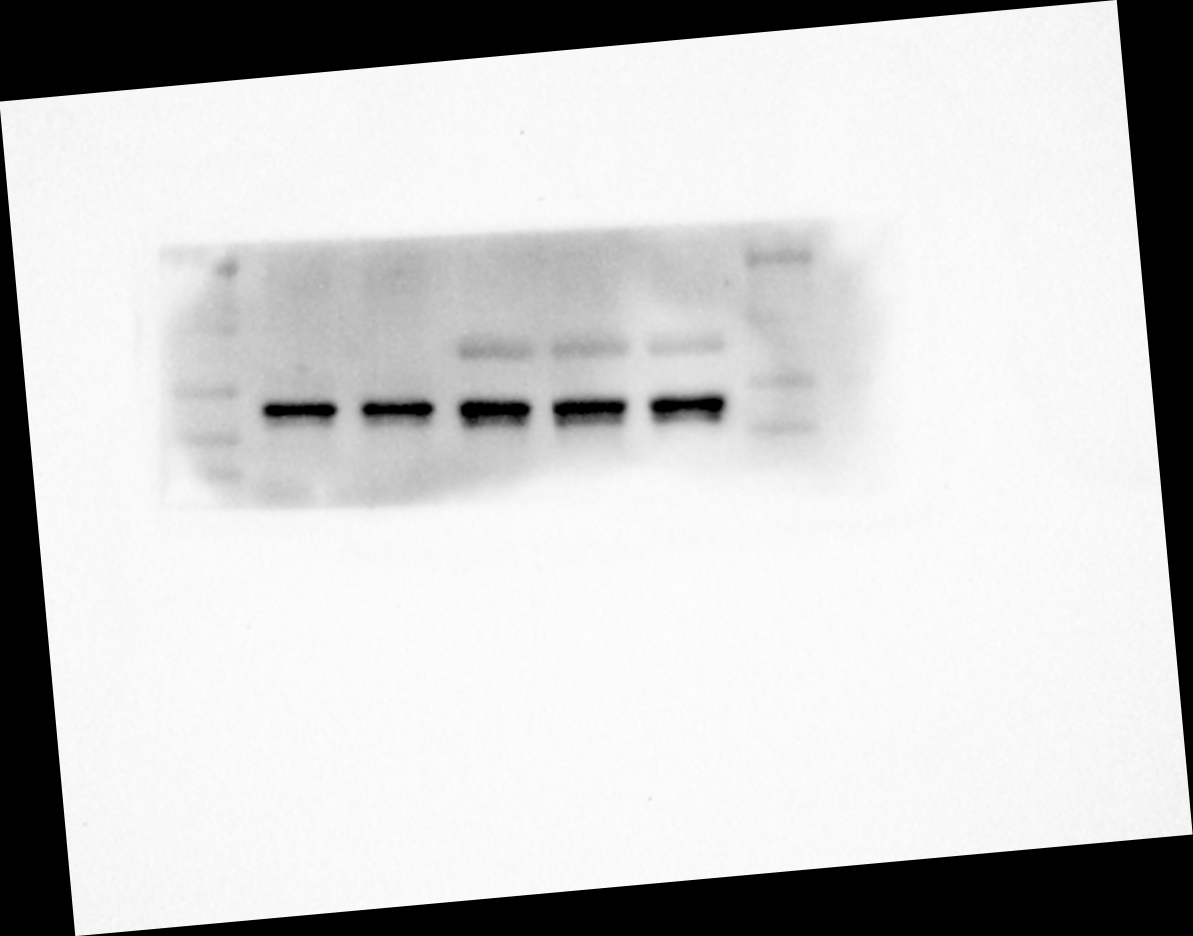

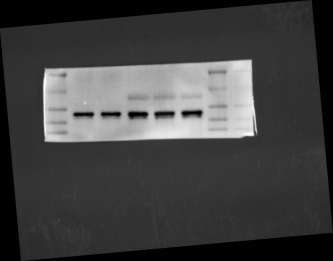

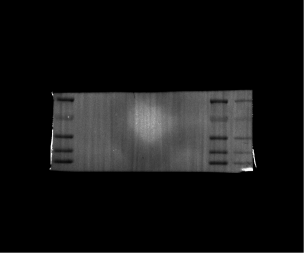


β-actin β-actin +marker marker


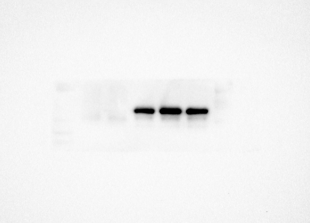

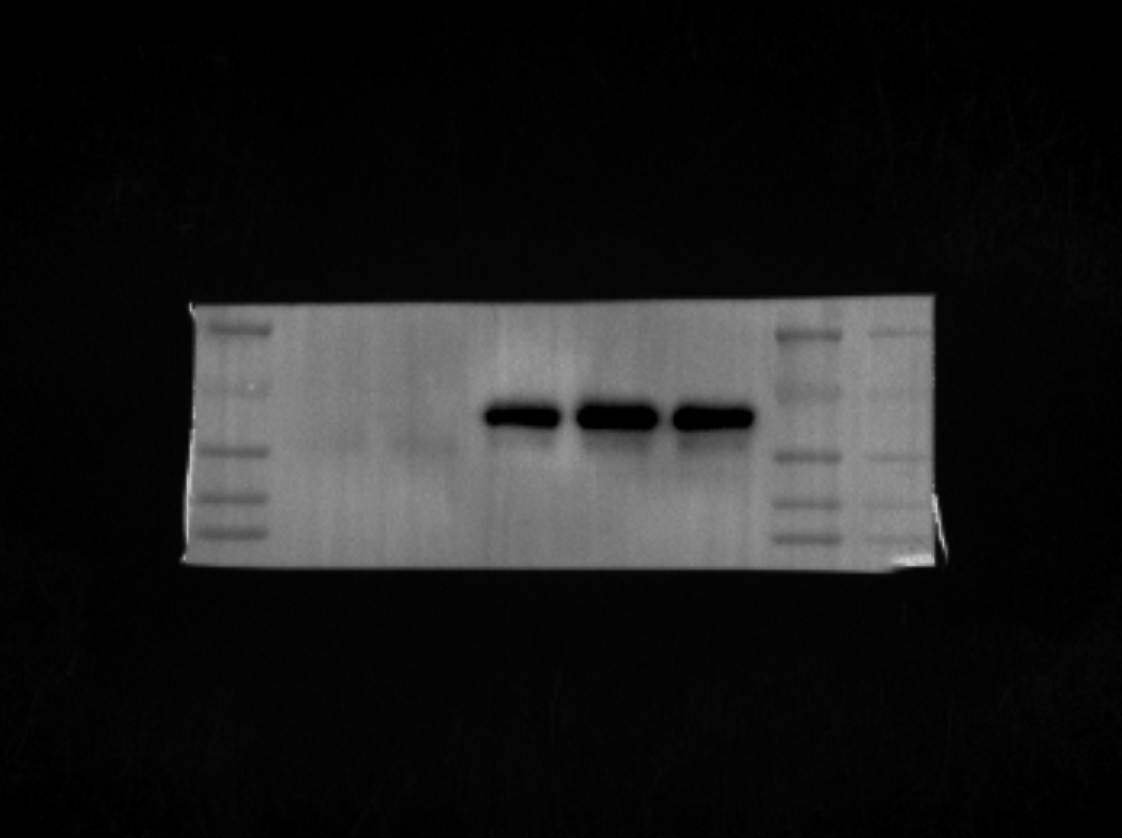


IKZF-1 IKZF-1+marker

**Group 3**

**
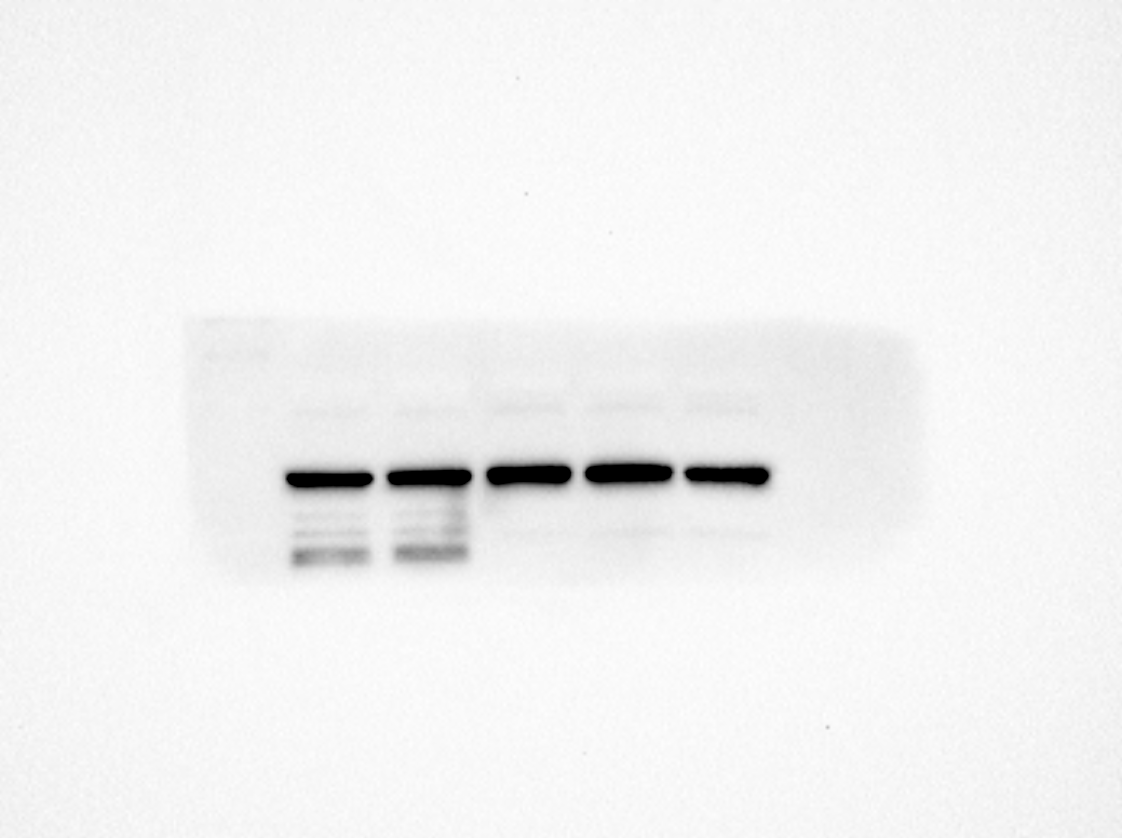

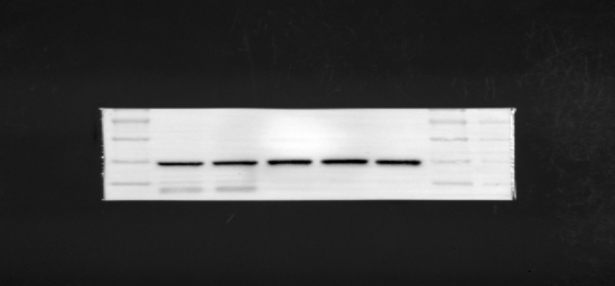

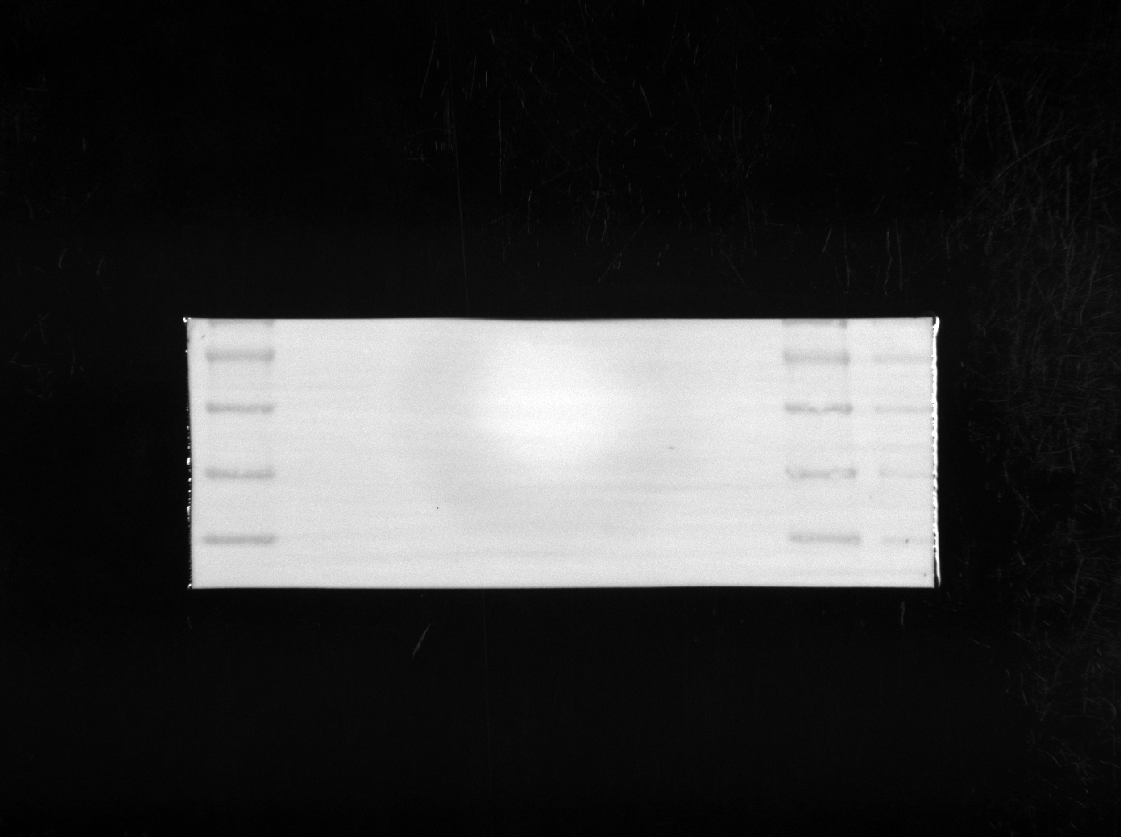
**

β-actin β-actin +marker marker

**
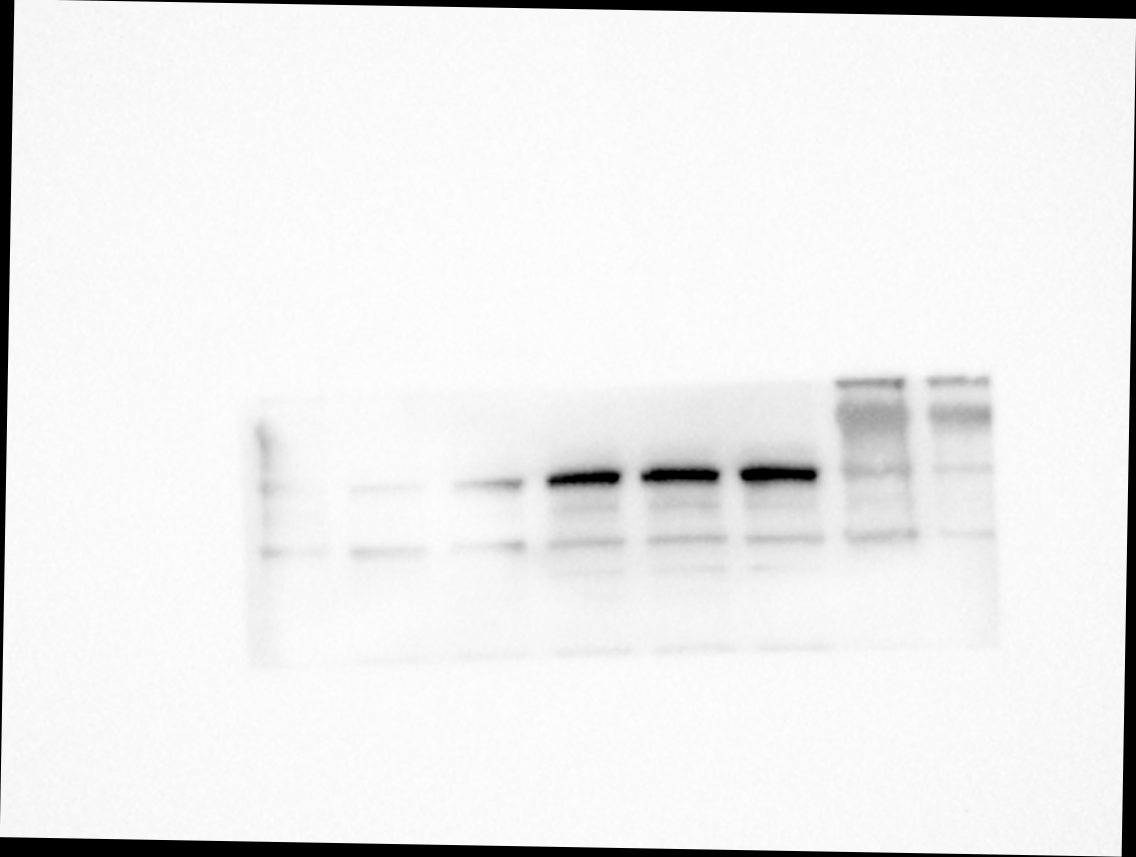

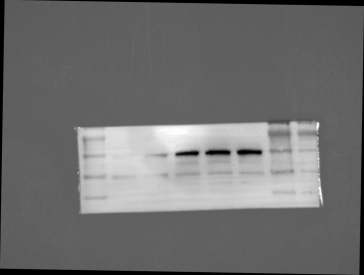
**

IKZF-1 IKZF-1+marker

**②Periodontitis+WAS(Target protein):**

**Group 1**

**
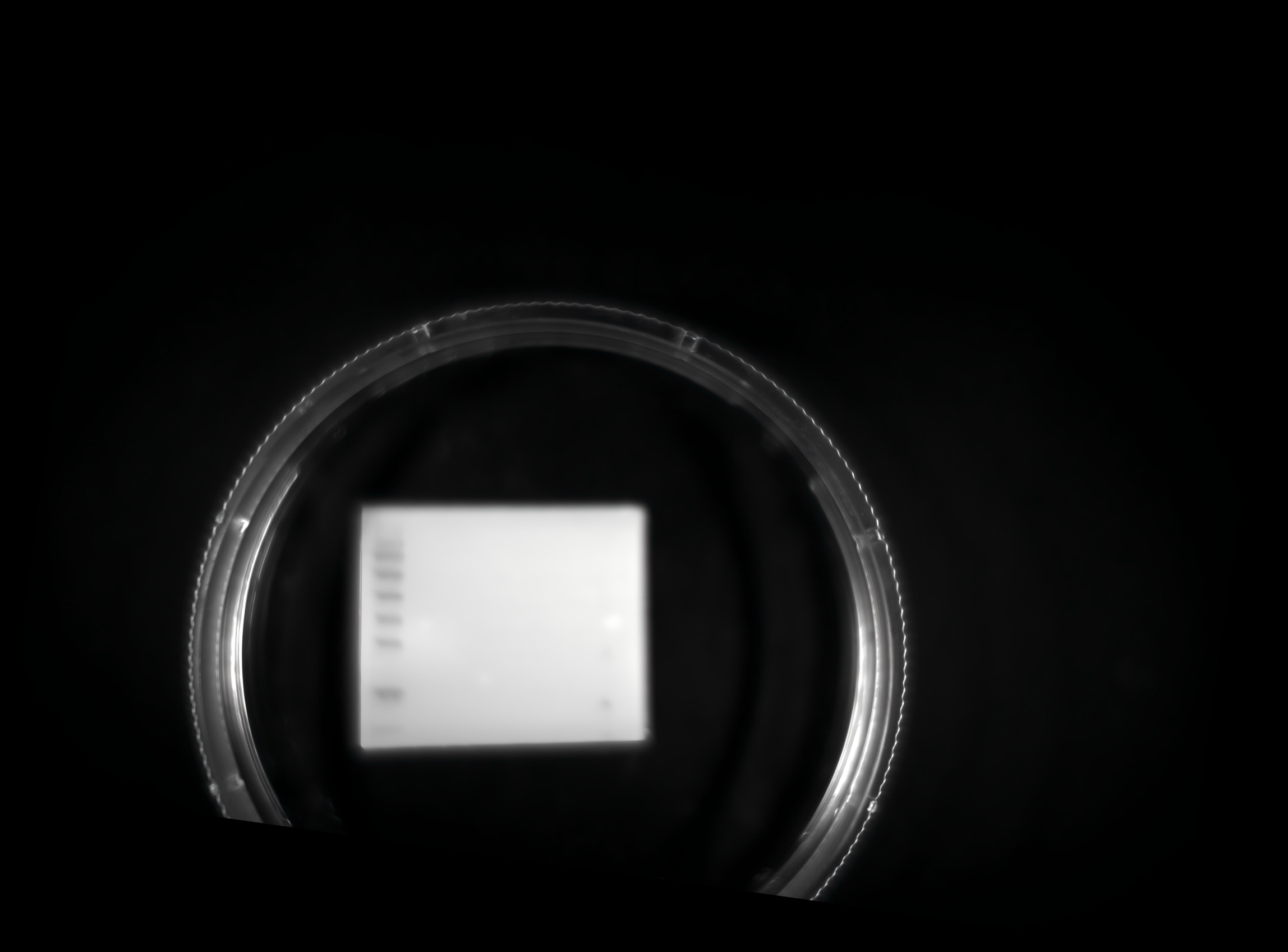

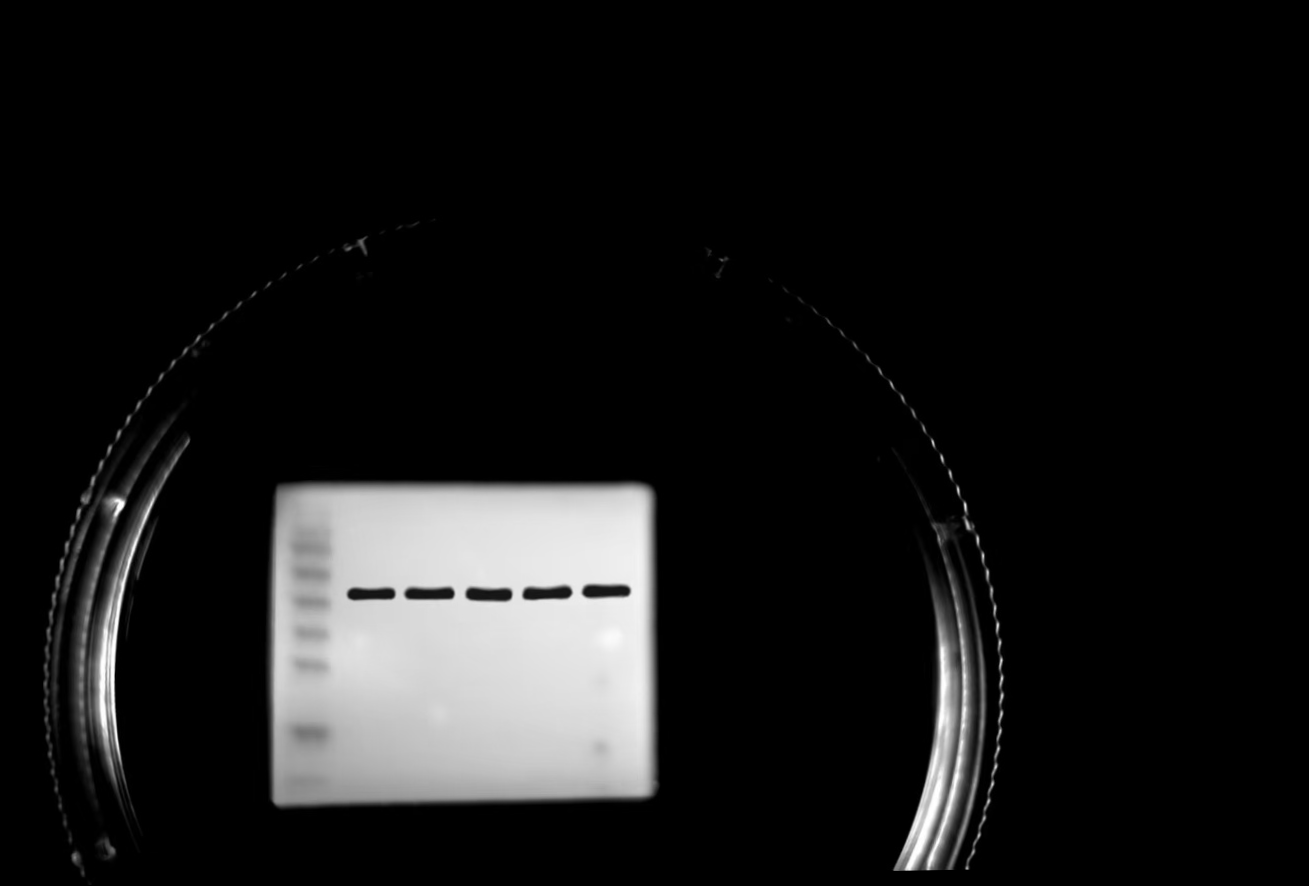
**

Marker β-actin +marker


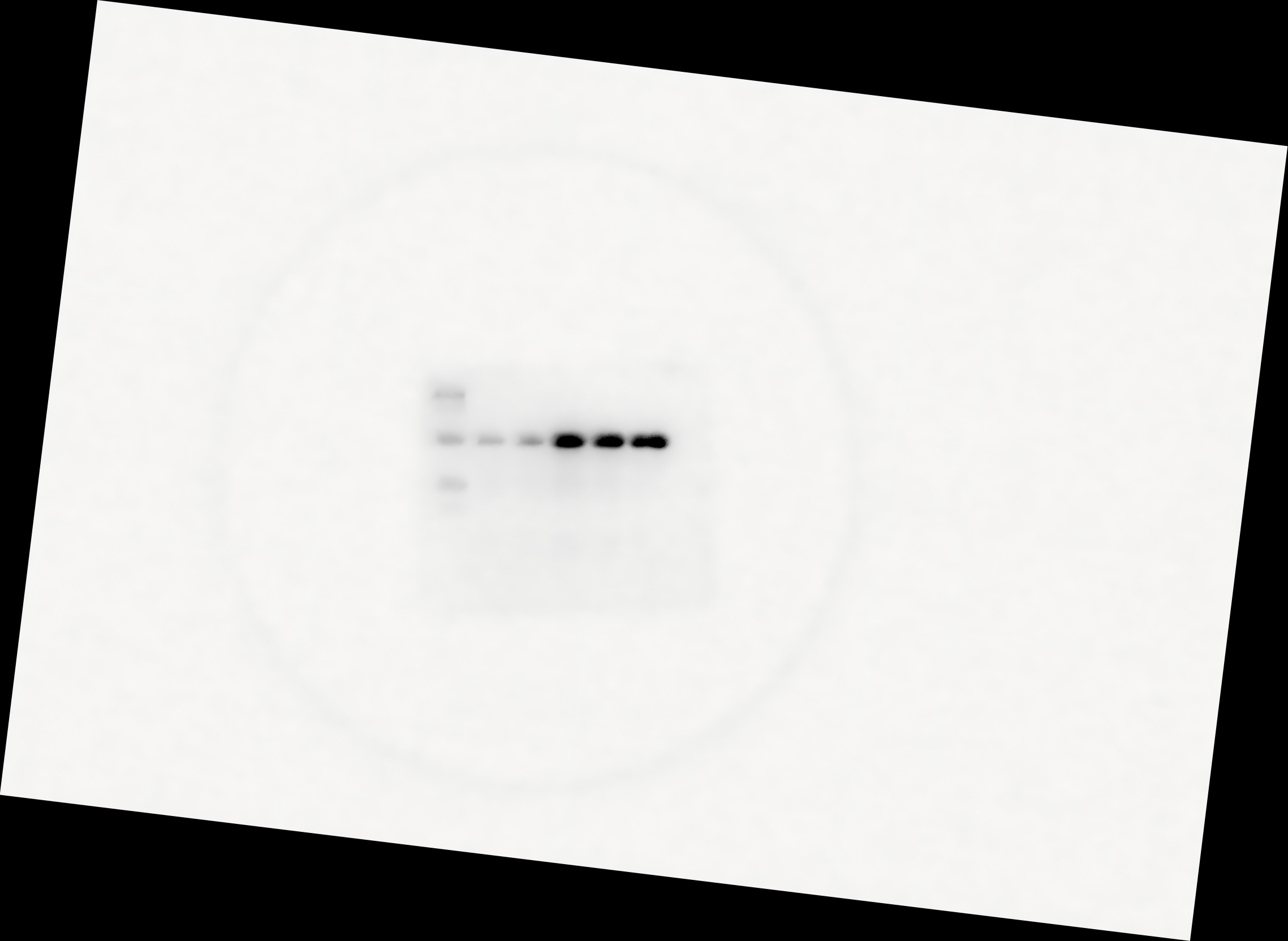

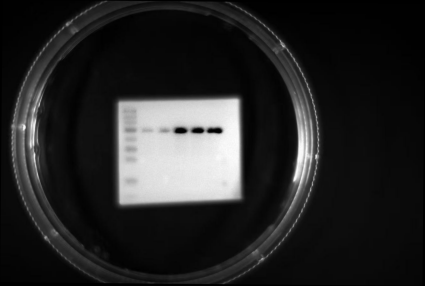


WAS WAS+marker

**Group 2**

**
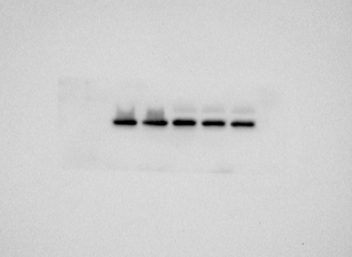

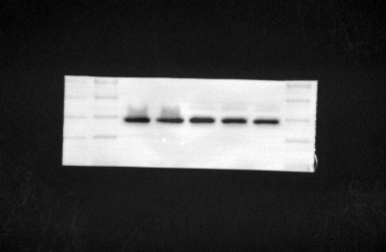

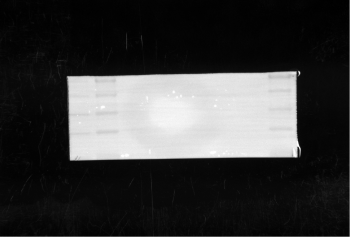
**

β-actin β-actin +marker marker

**
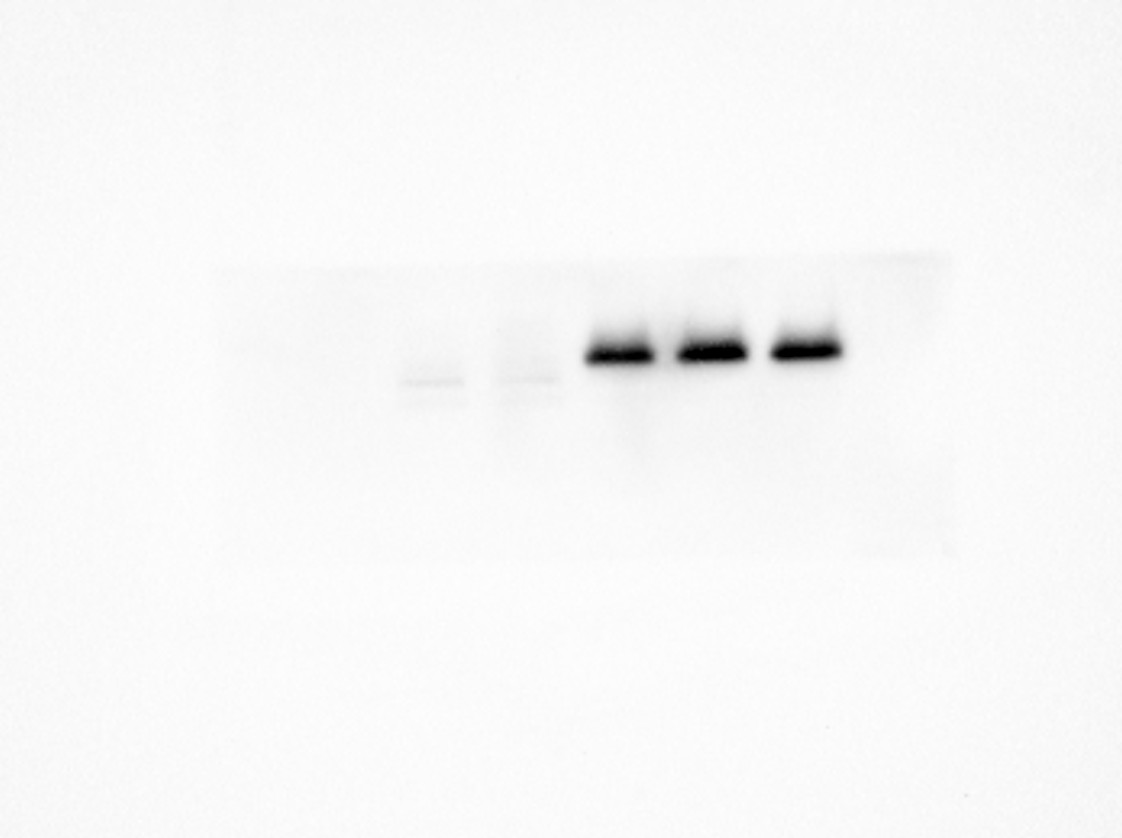

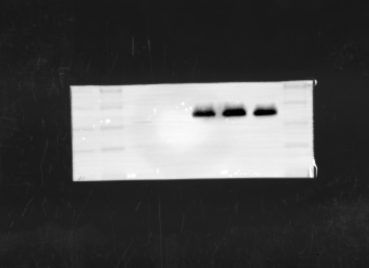
**

WAS WAS+marker

**Group 3**


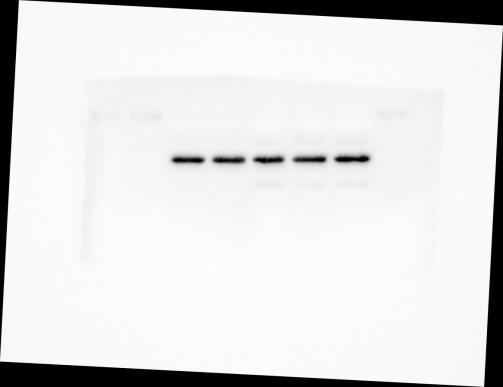

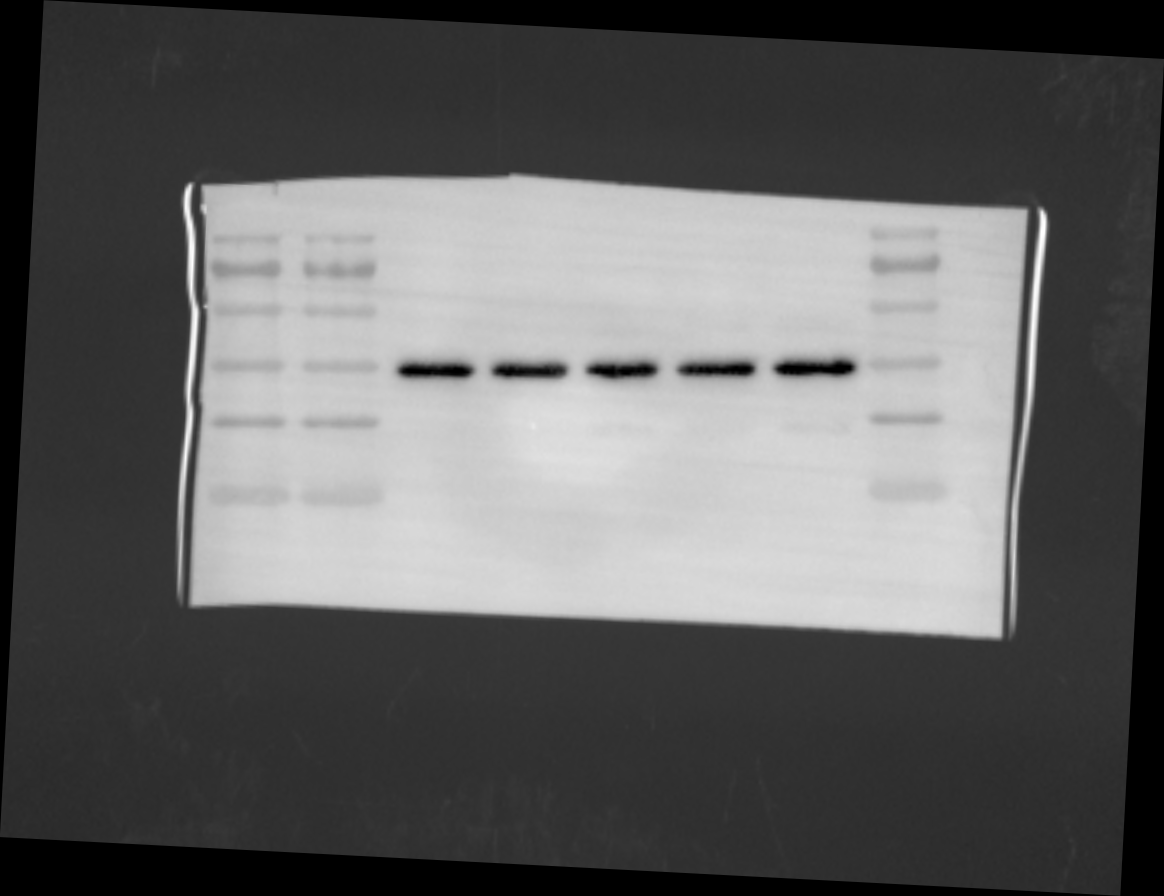


β-actin β-actin +marker


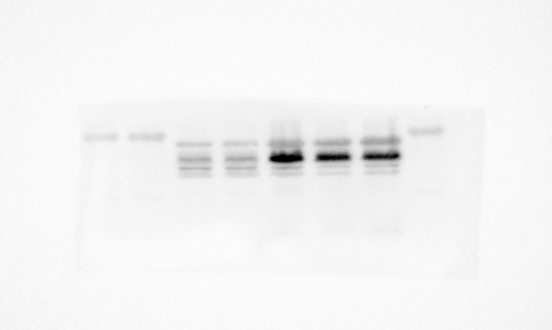

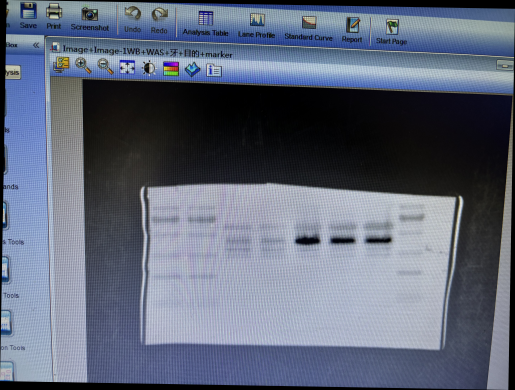


WAS WAS+marker

**③RCC+IKZF-1(Target protein):**

**Group 1**

**
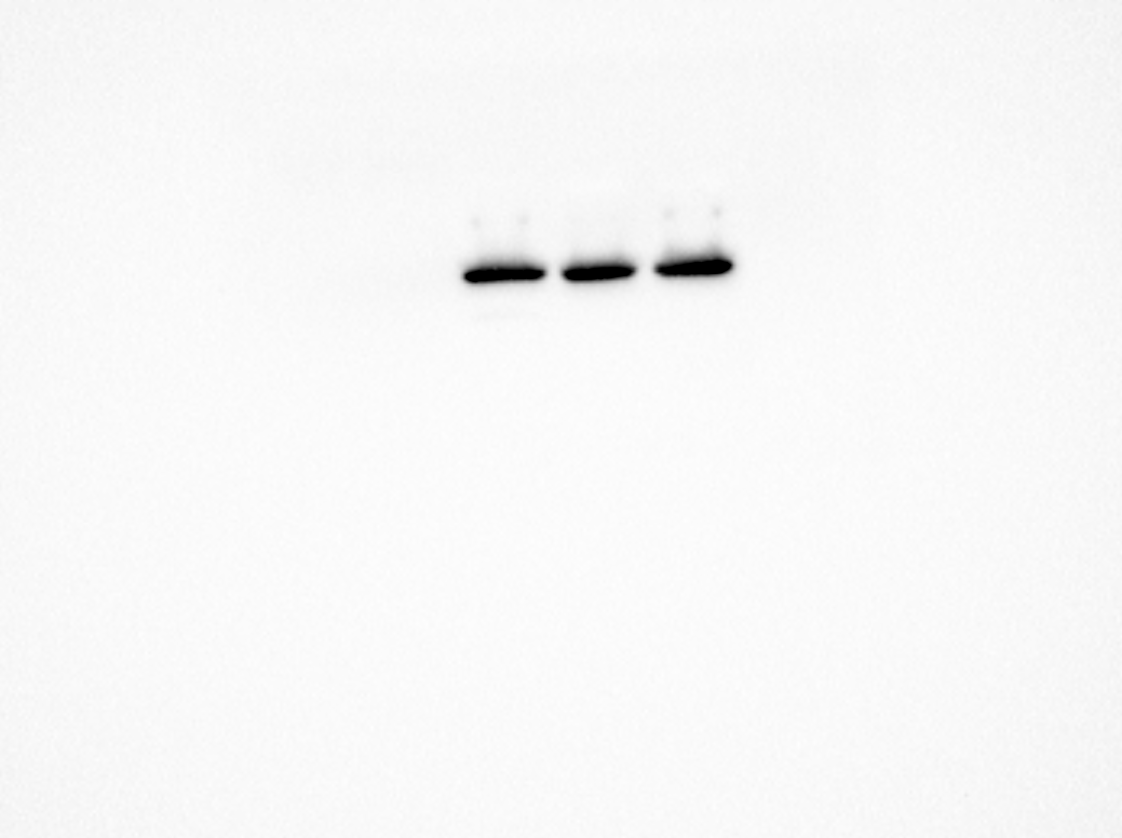

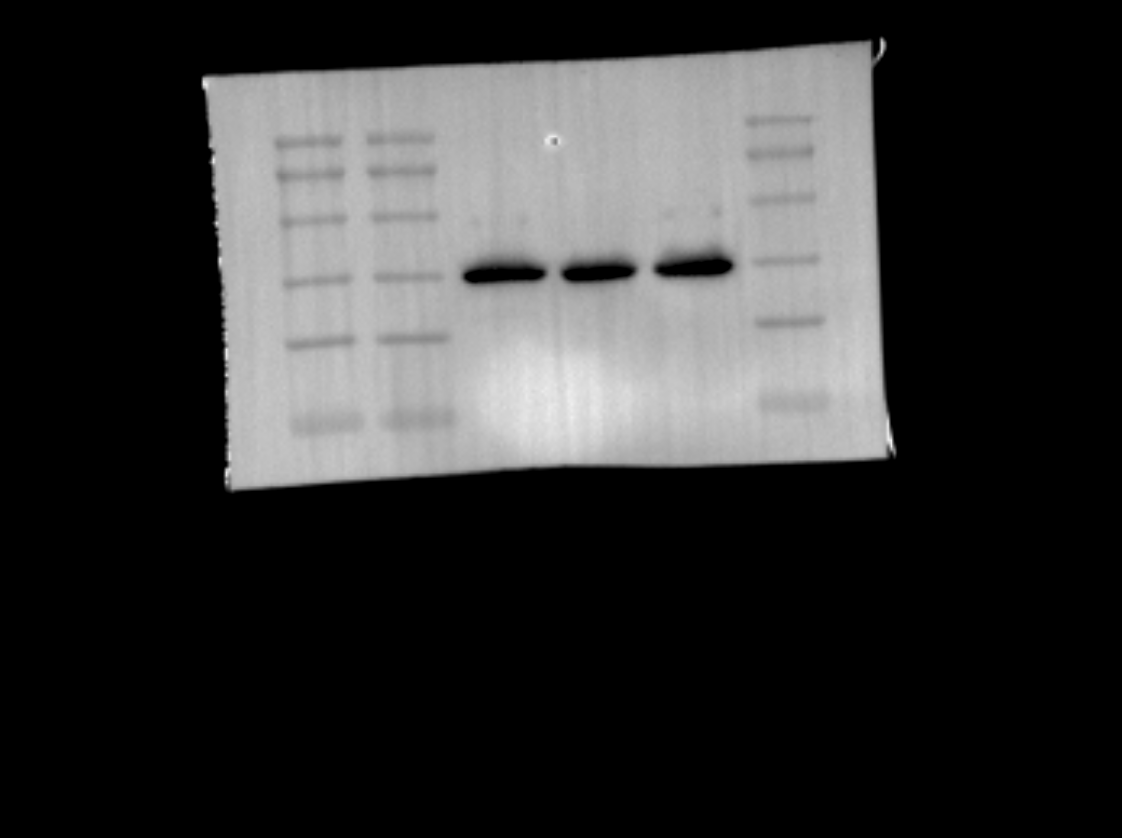
**

β-actin β-actin +marker

**
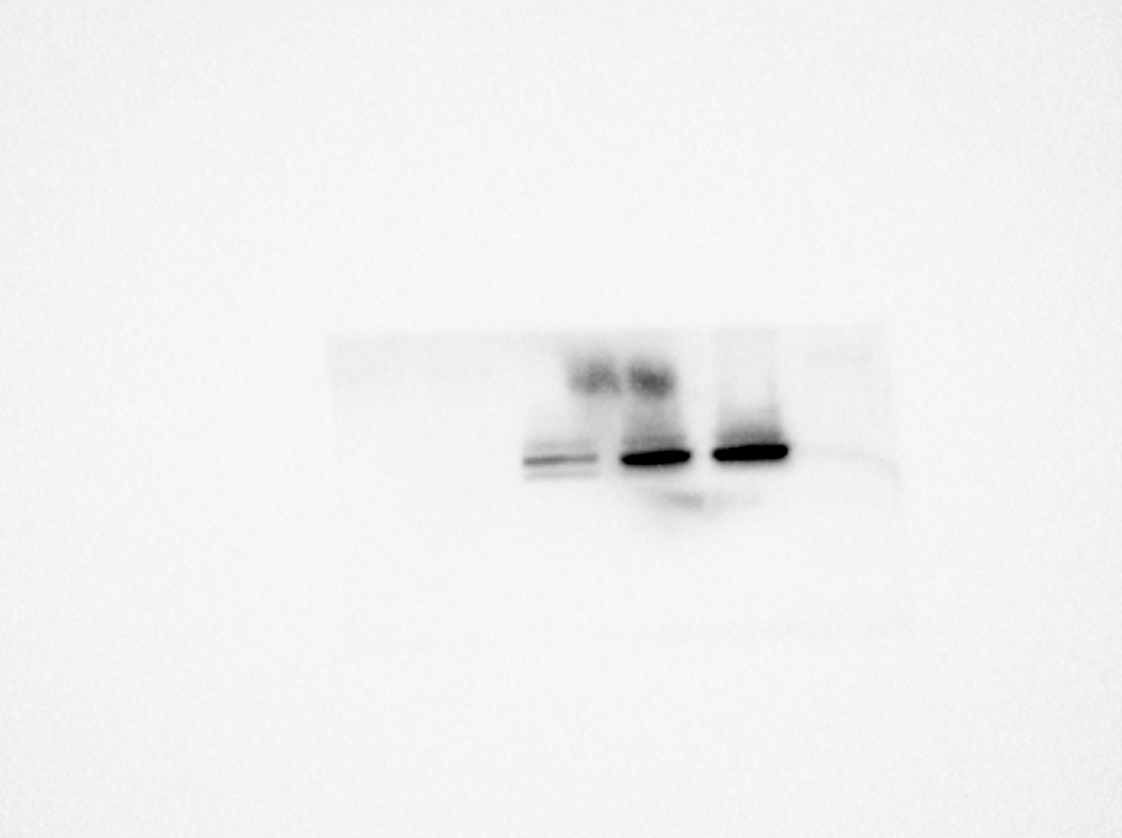

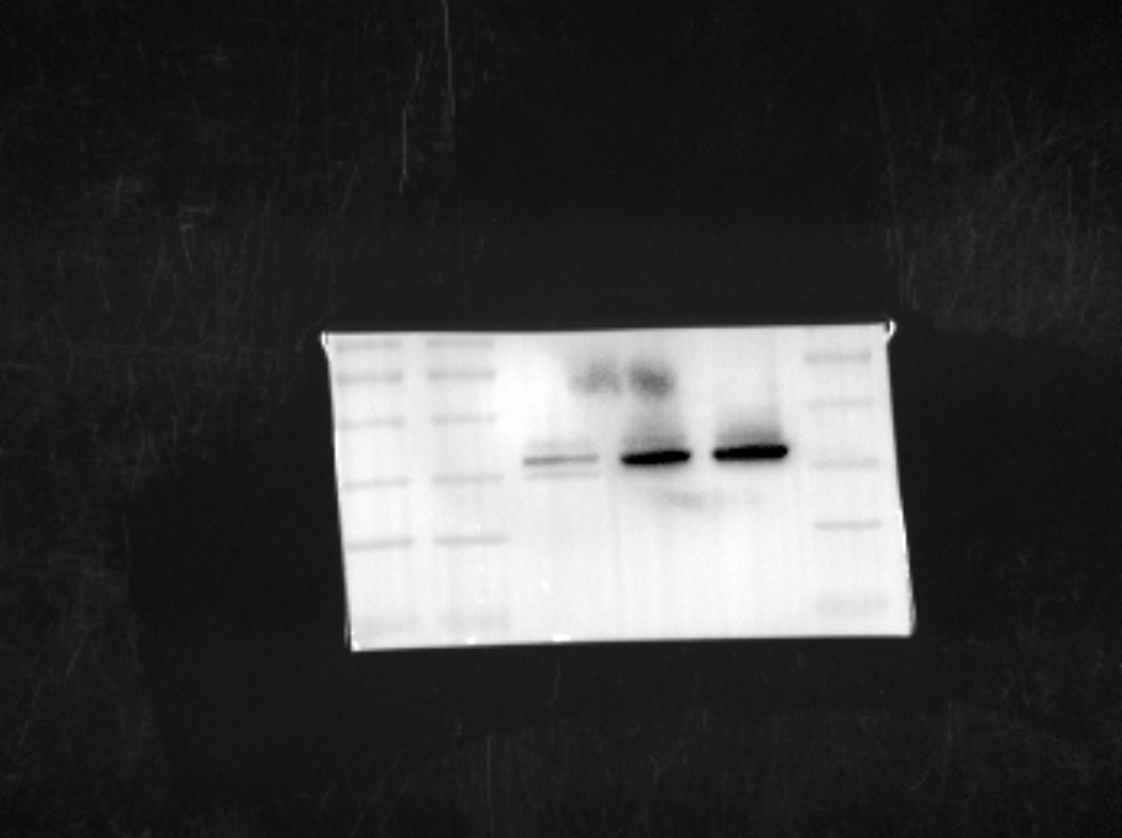
**

IKZF-1 IKZF-1+marker

**Group 2**

**
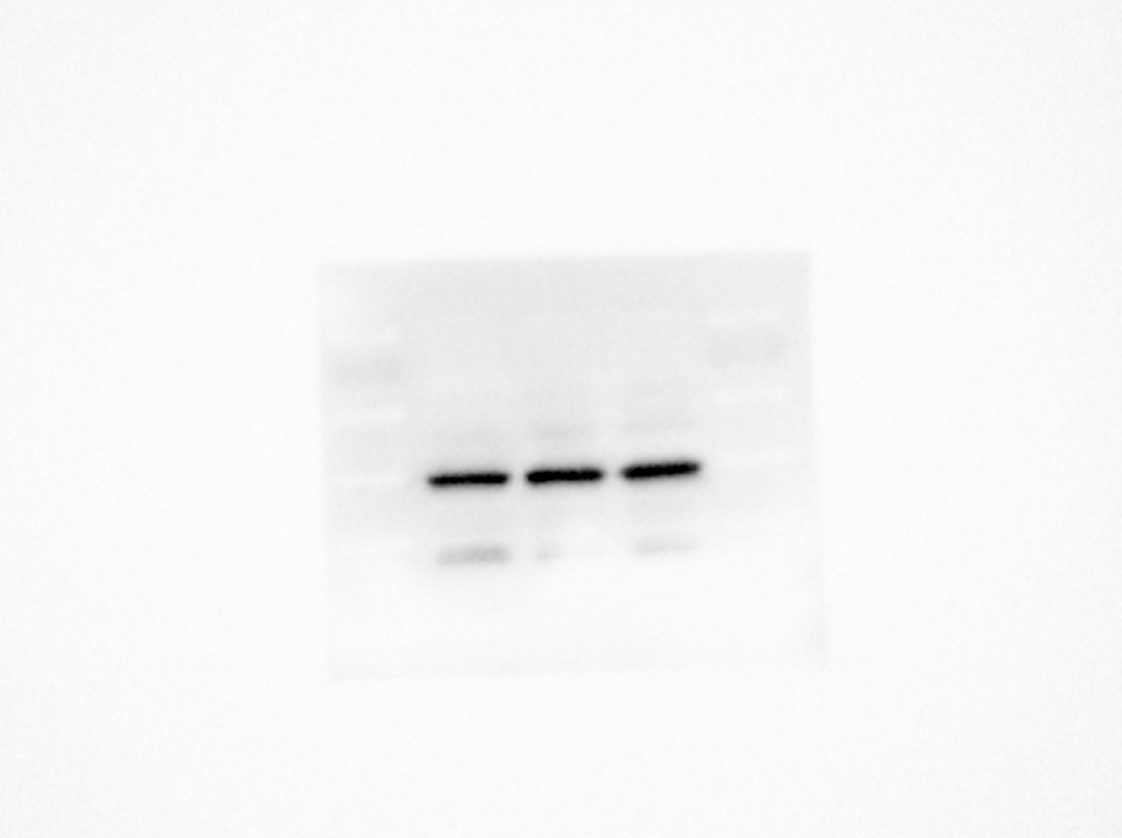

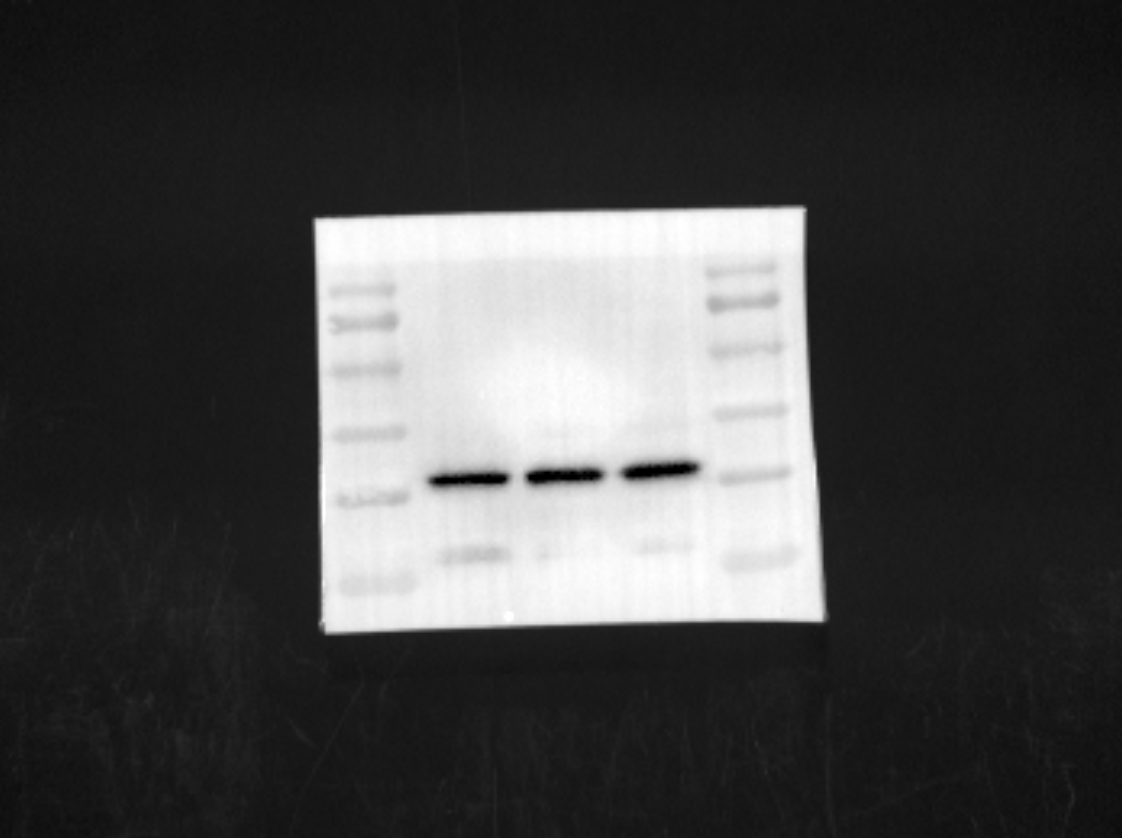
**

β-actin β-actin +marker

**
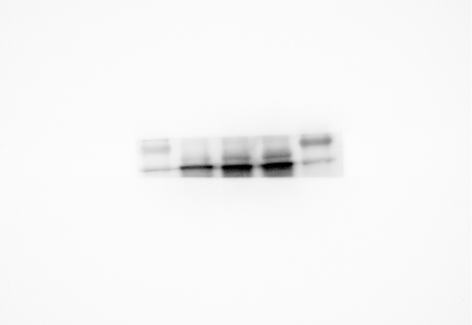

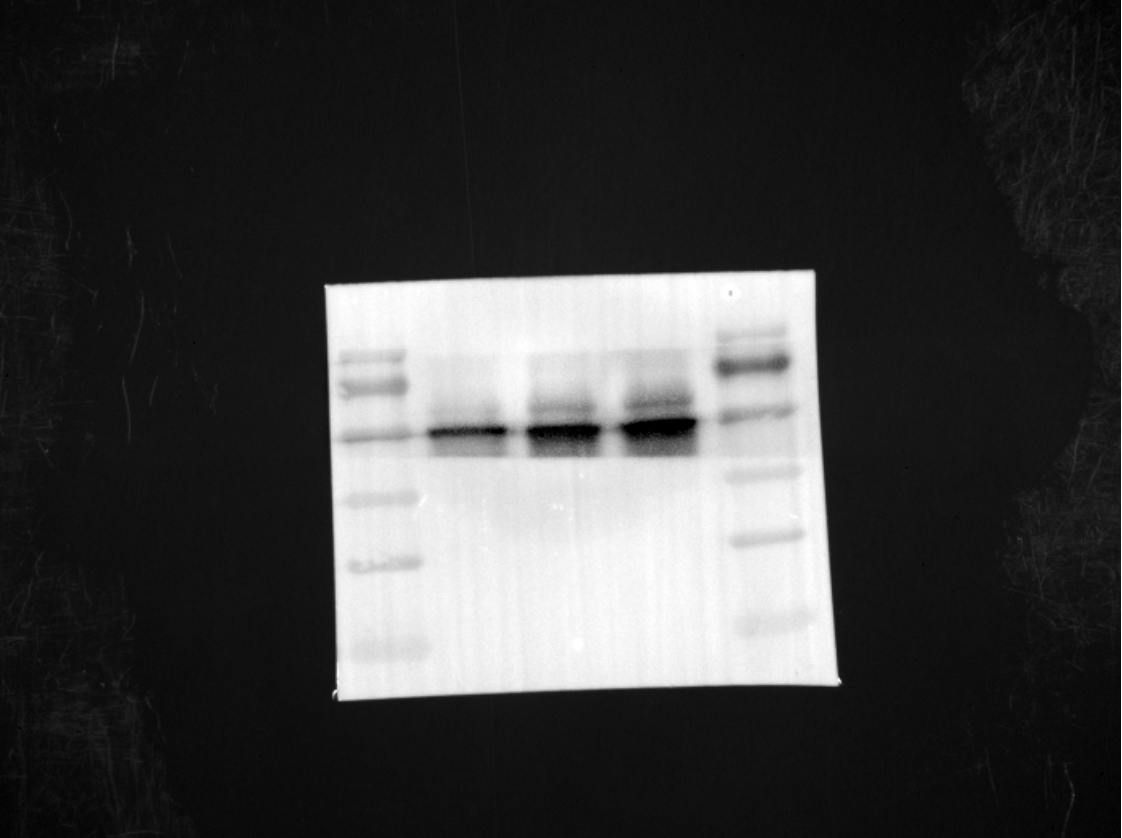
**

IKZF-1 IKZF-1+marker

**Group 3**

**
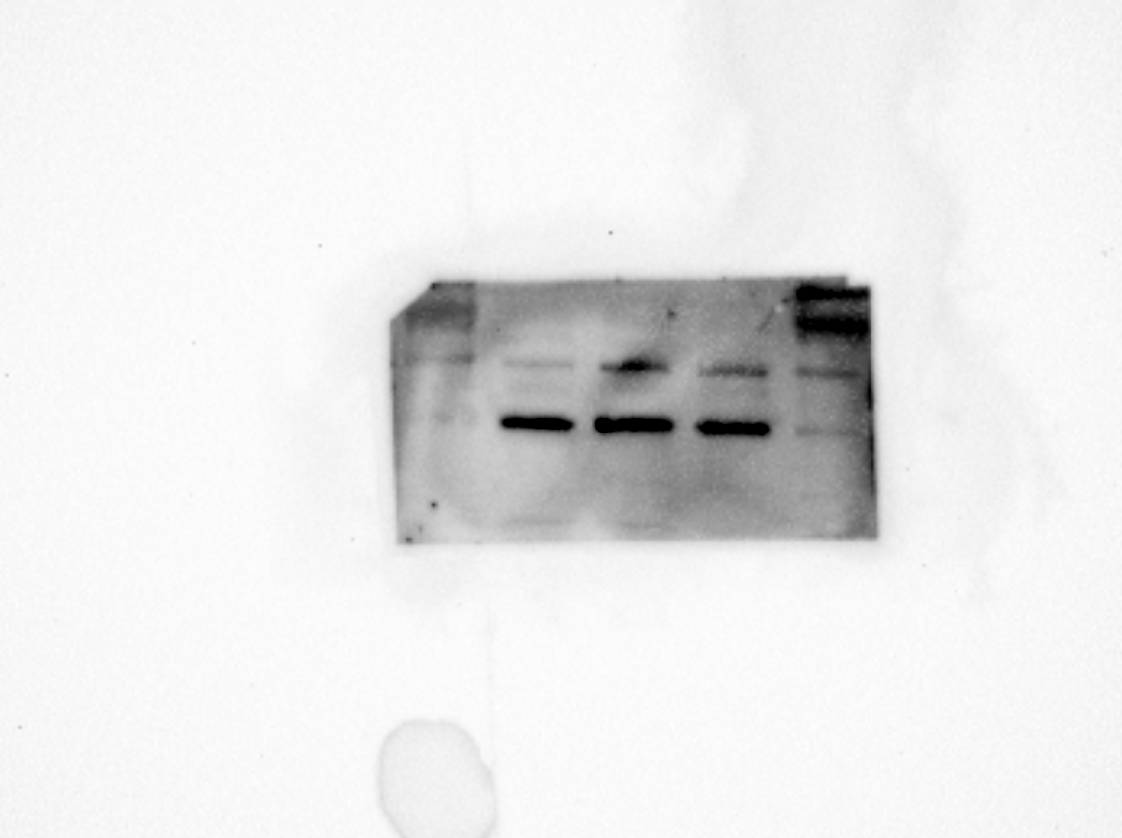

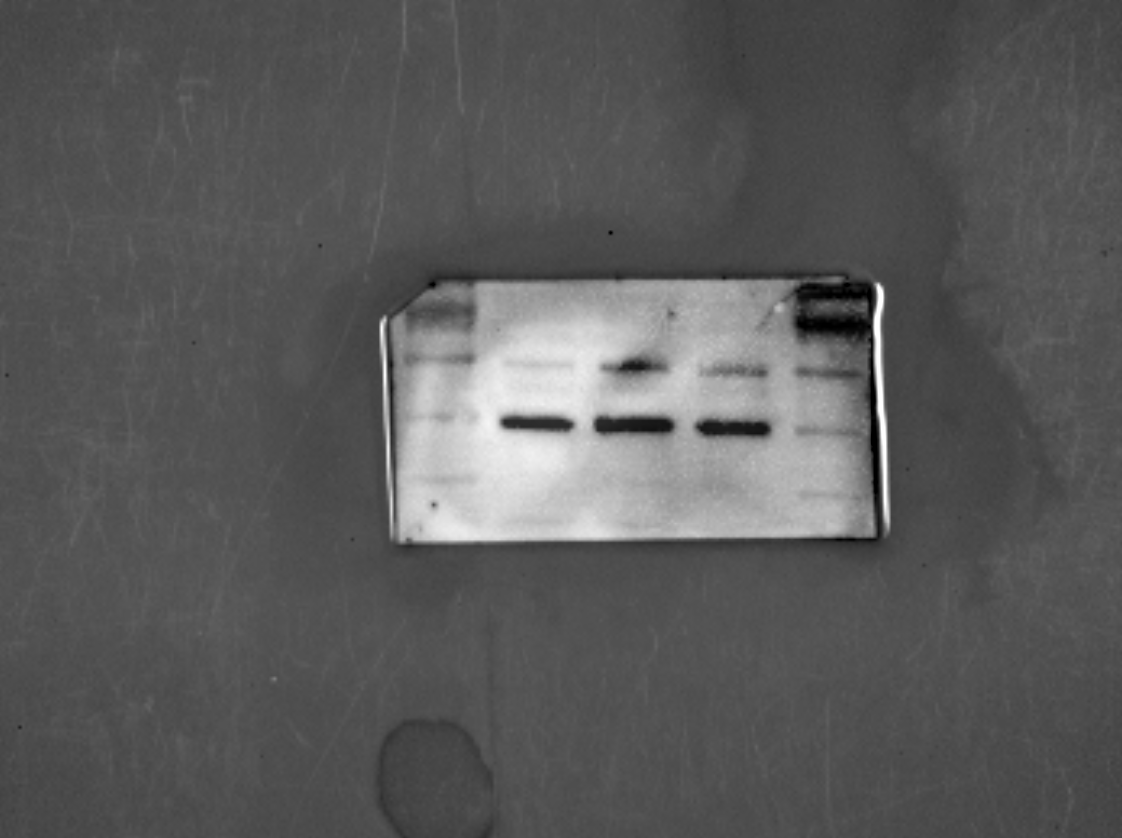

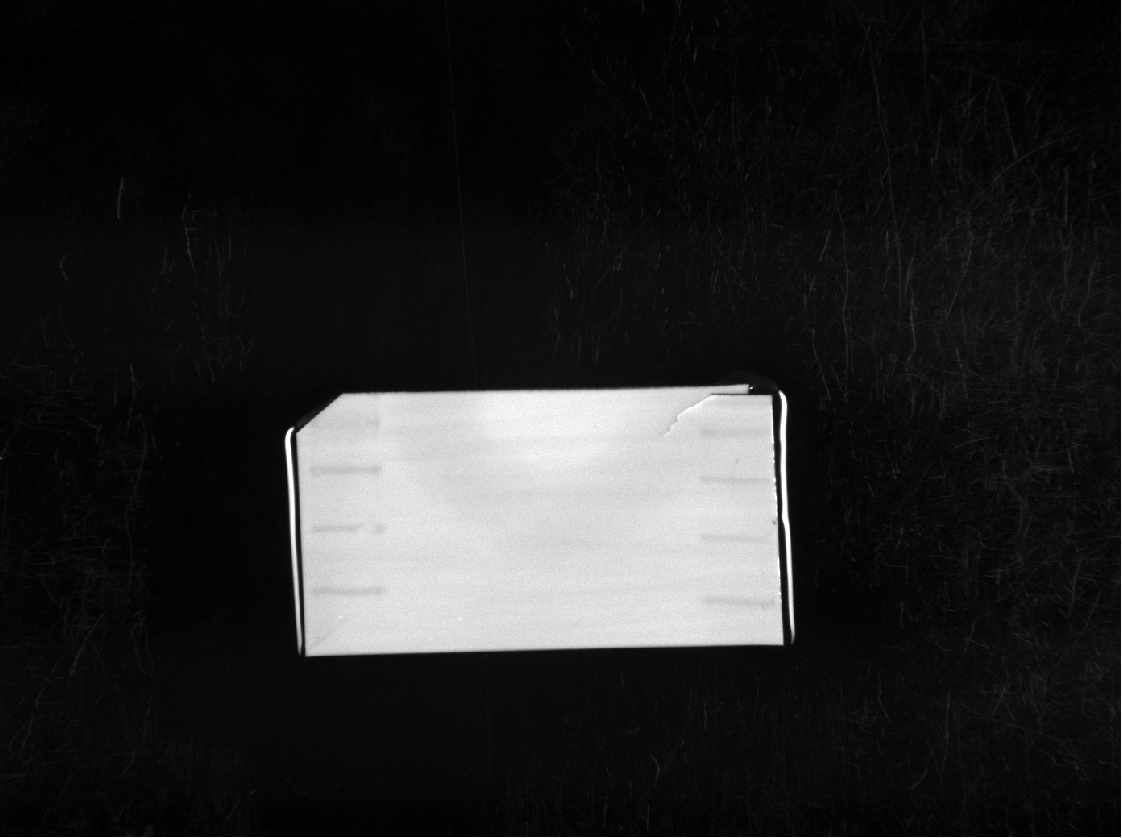
**

β-actin β-actin +marker marker

**
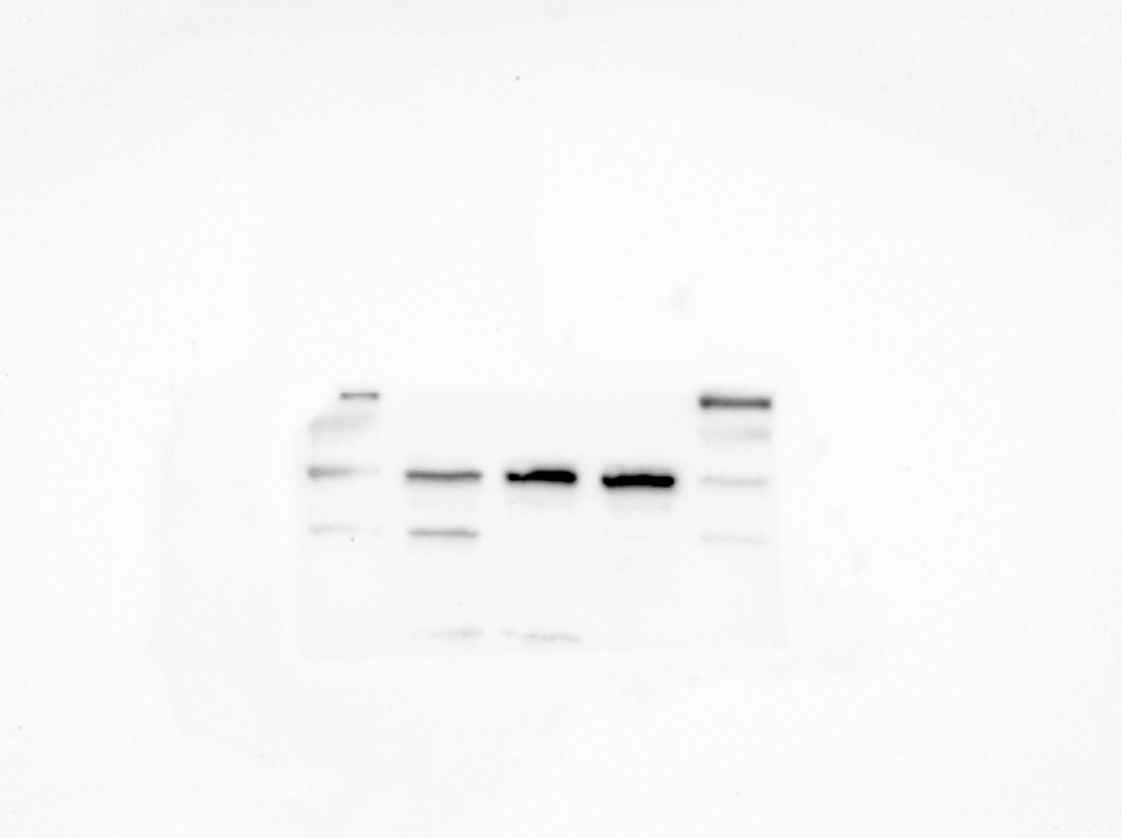

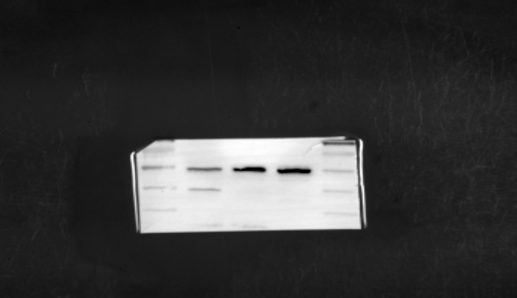
**

IKZF-1 IKZF-1+marker

**④RCC+WAS(Target protein):**

**Group 1**

**
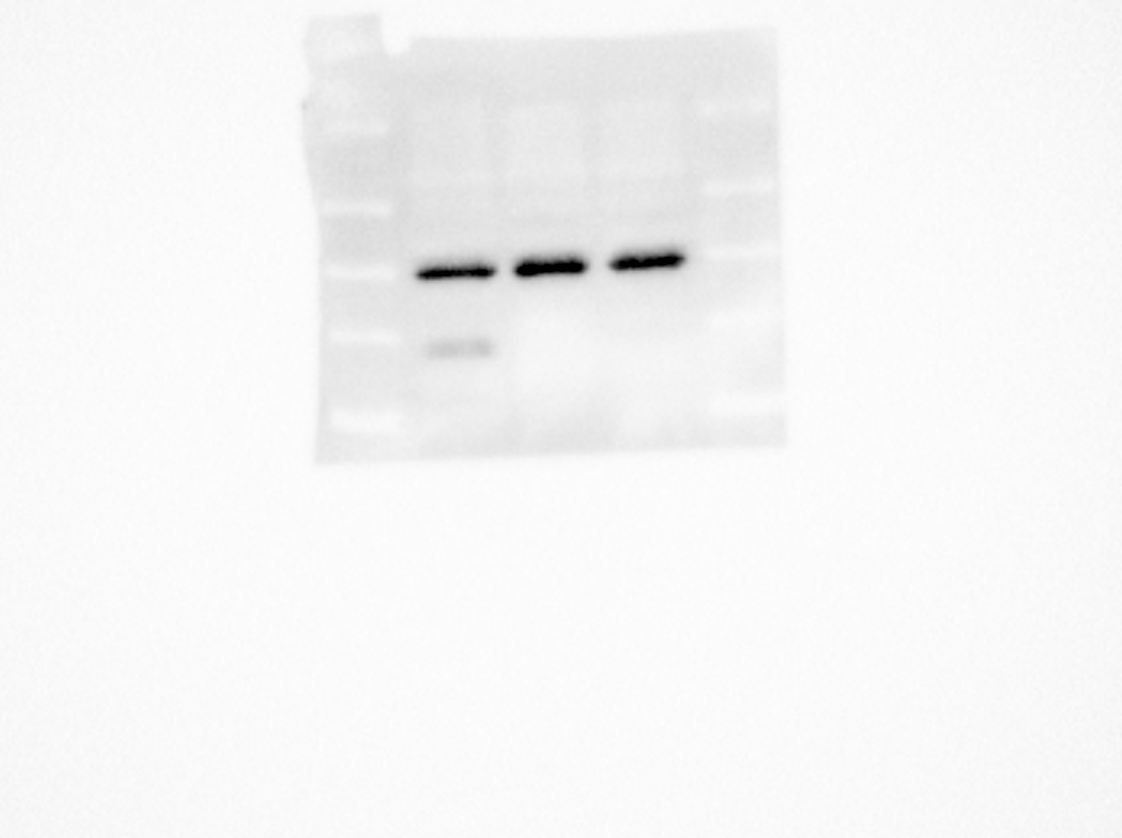

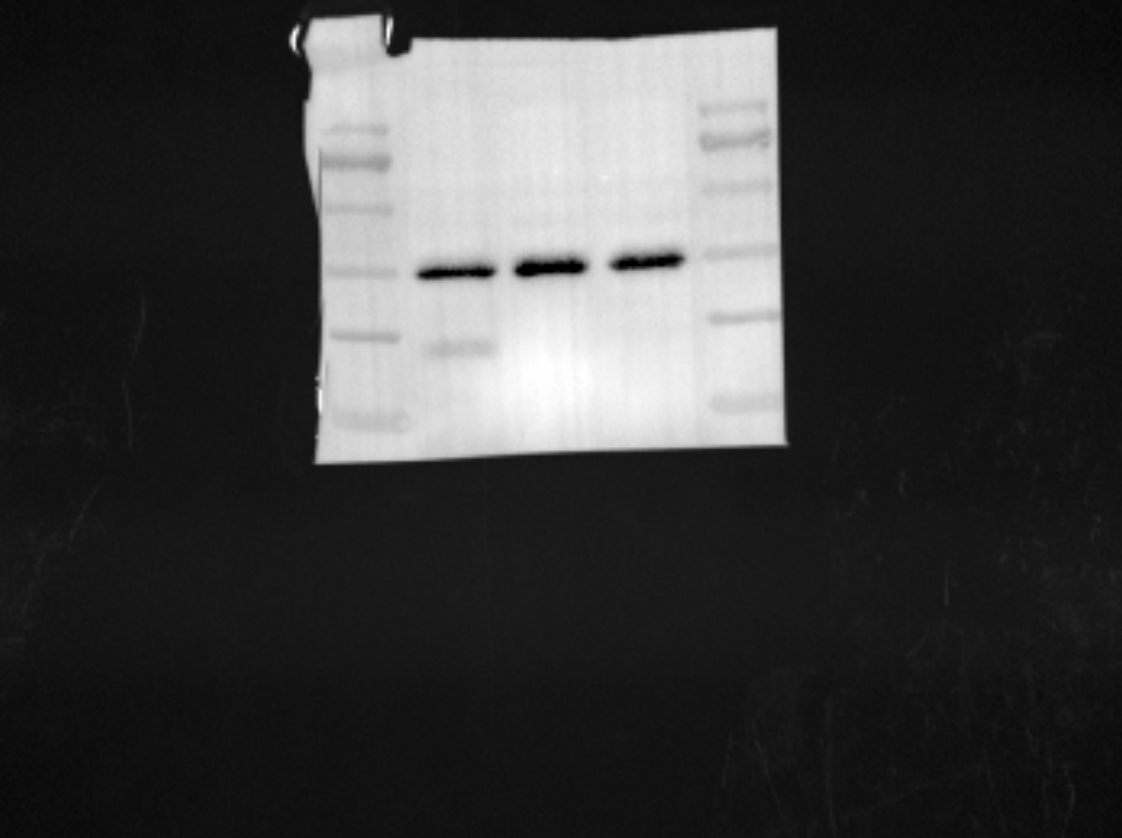
**

β-actin β-actin +marker

**
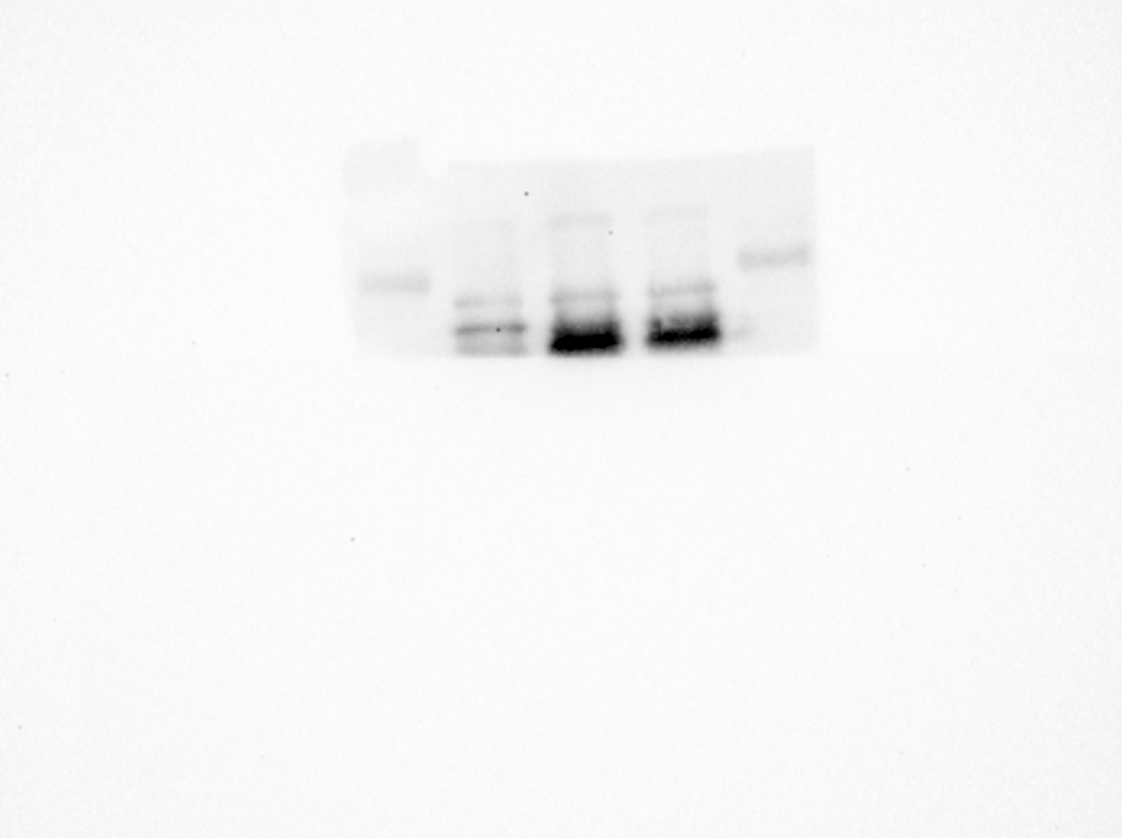

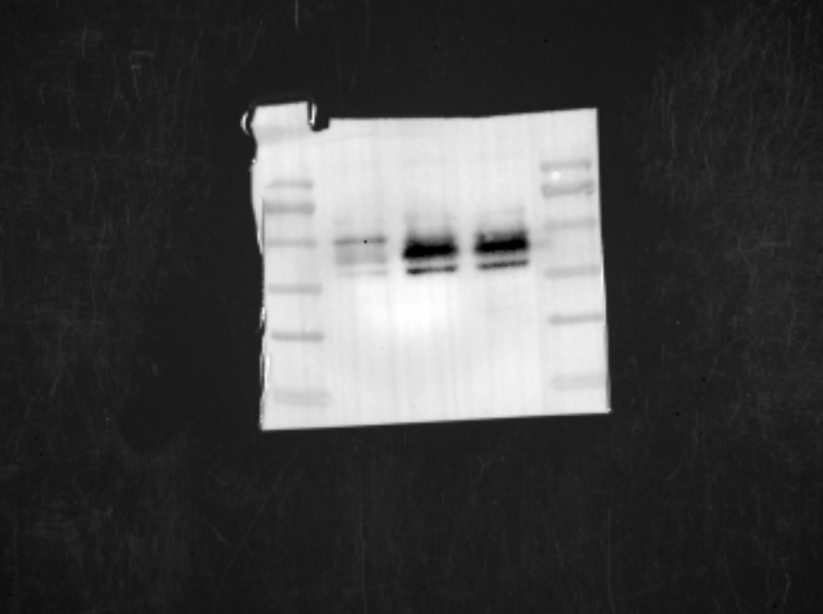
**

WAS WAS+marker

**Group 2**

**
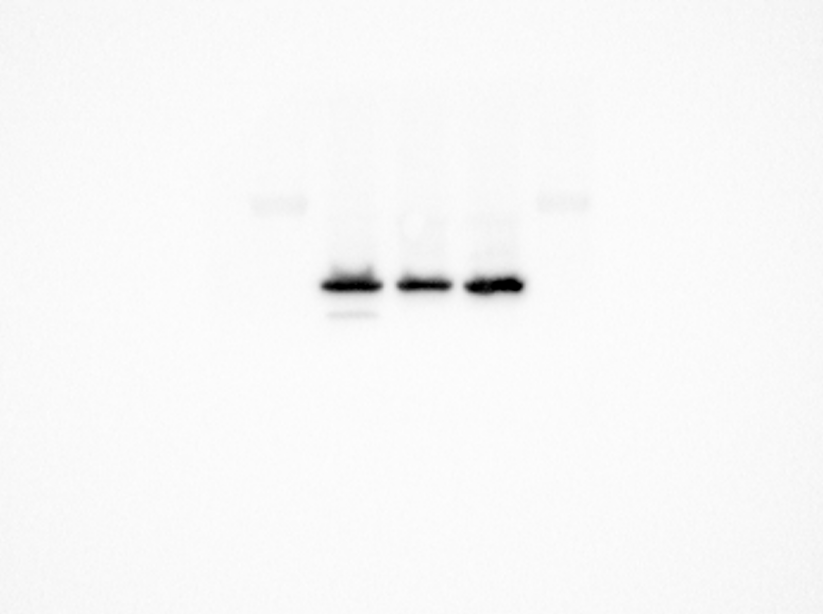

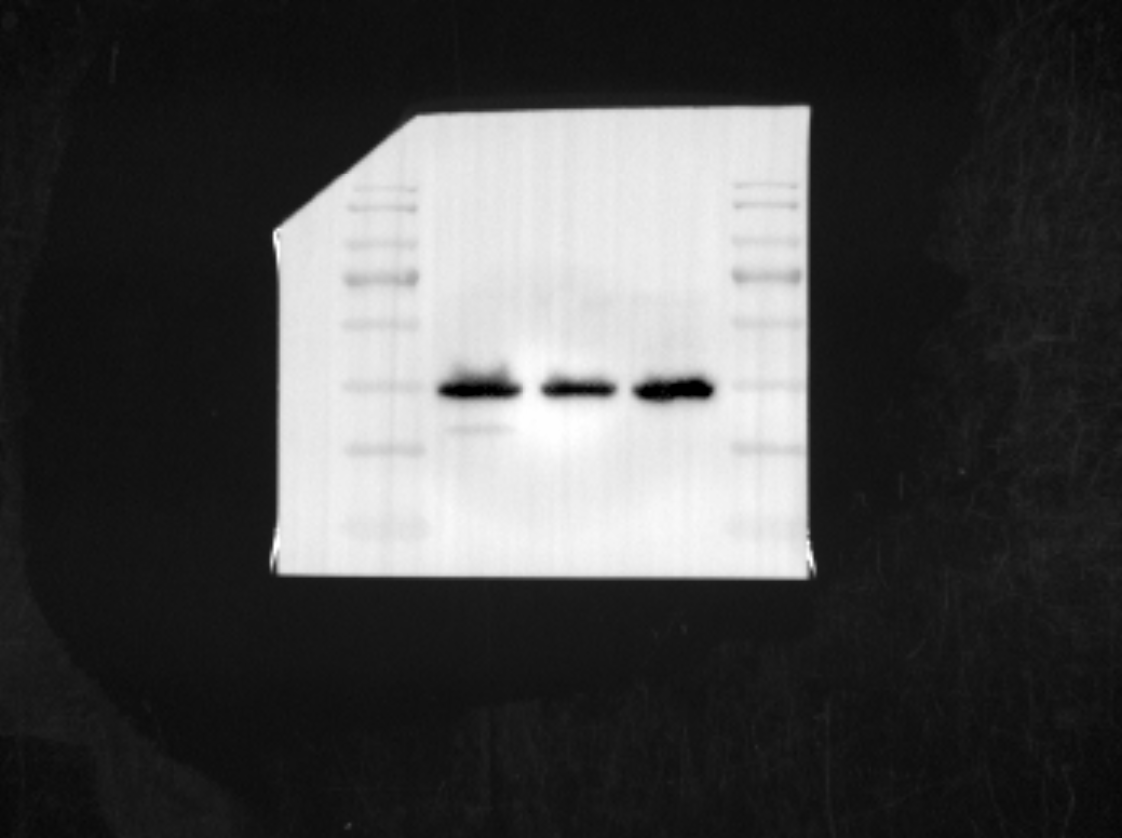
**

β-actin β-actin +marker

**
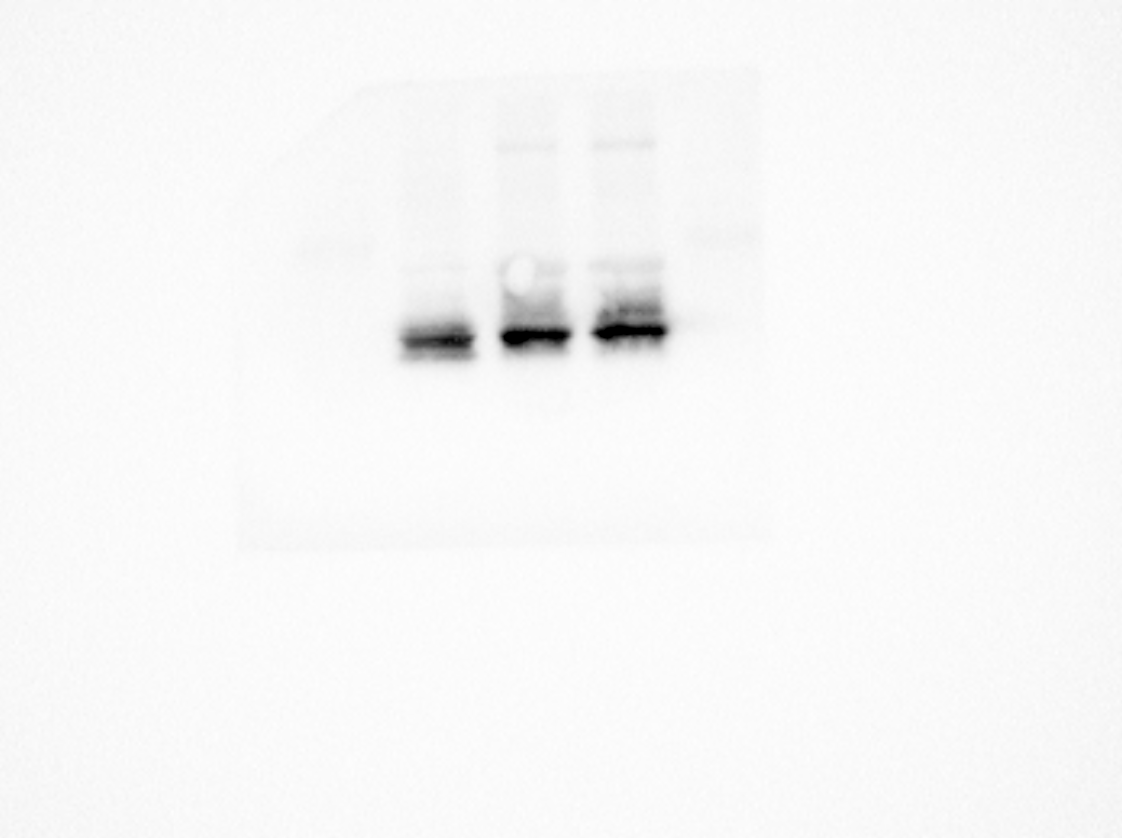

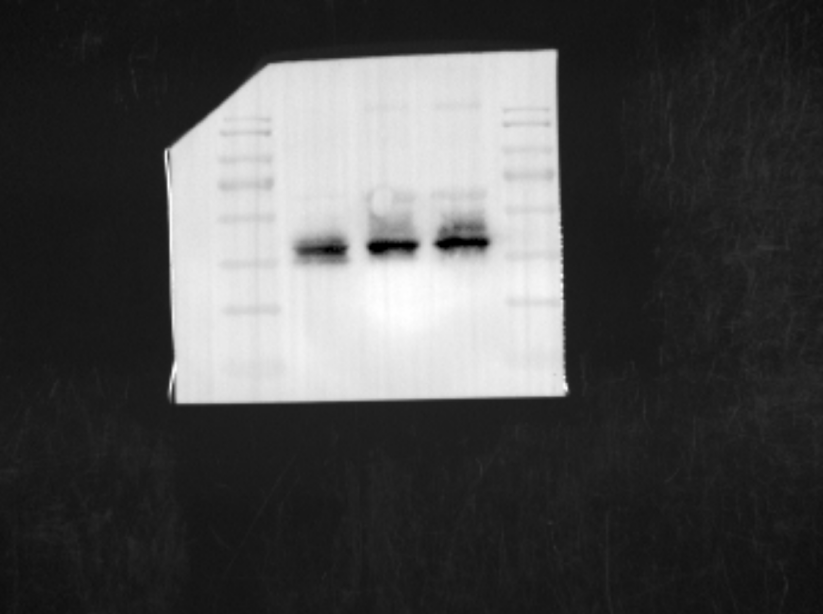
**

WAS WAS+marker

**Group 3**

**
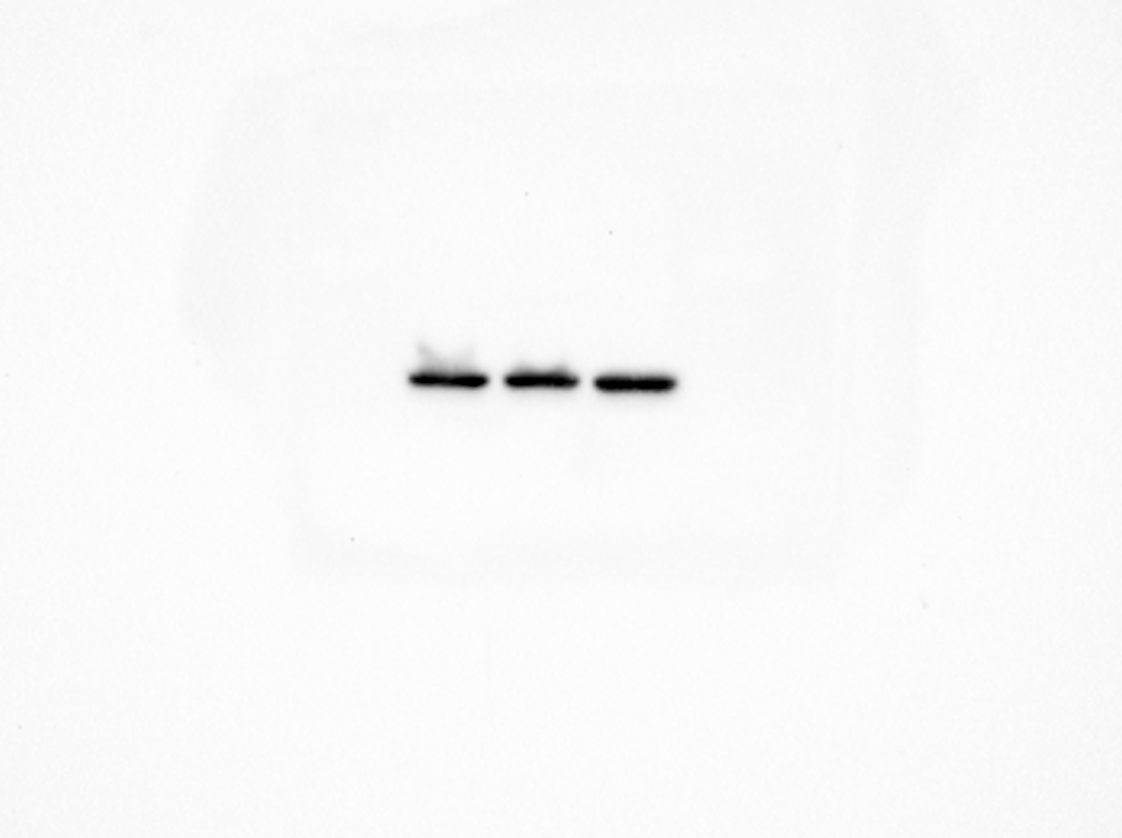

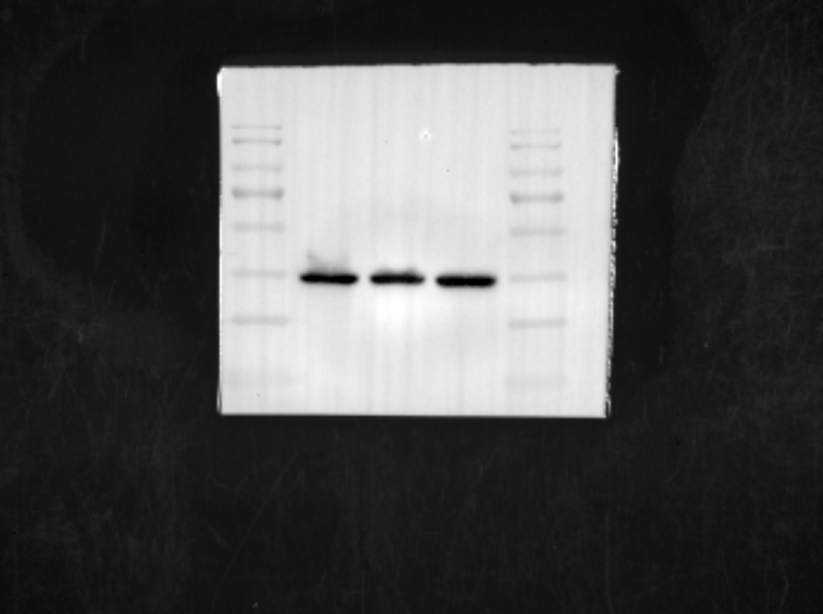
**

β-actin β-actin +marker

**
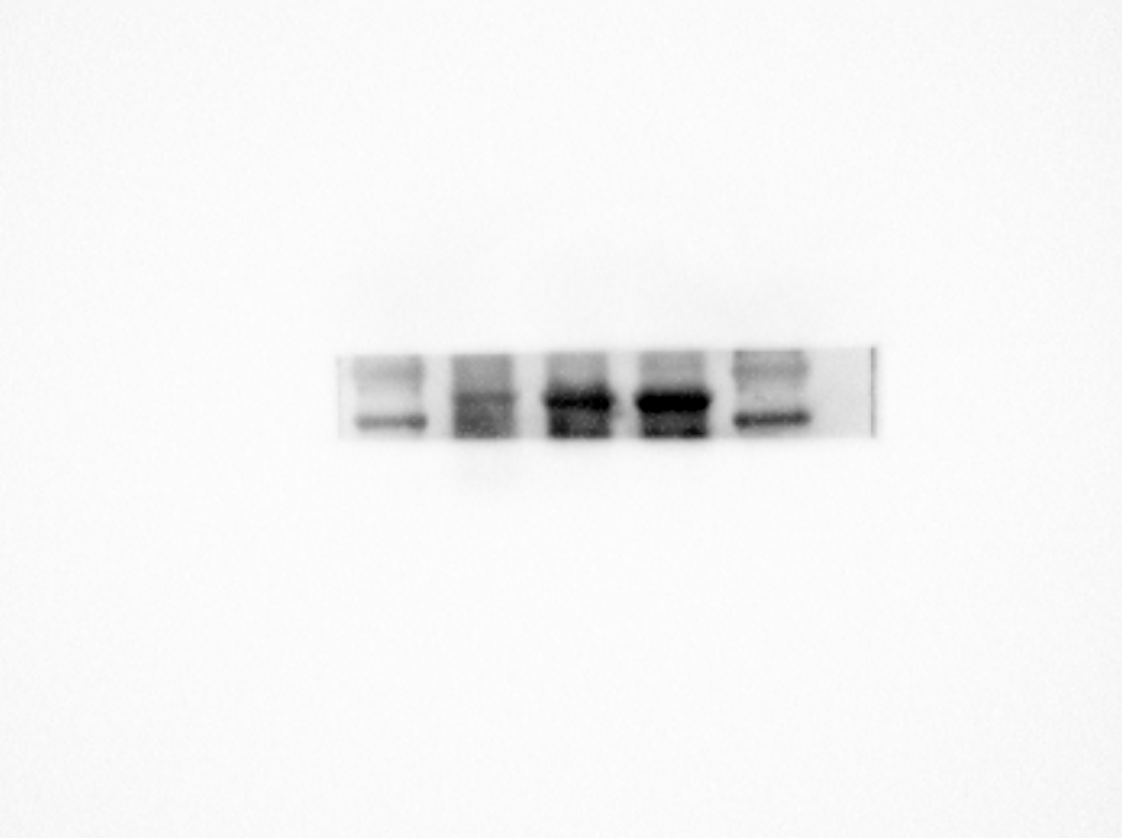

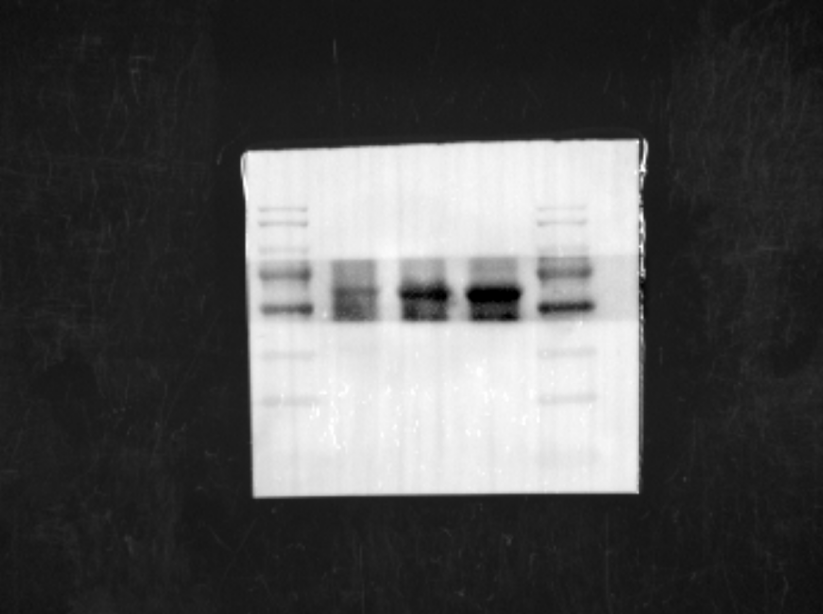
**

WAS WAS+marker
